# Population transcriptomic sequencing reveals allopatric divergence and local adaptation in *Pseudotaxus chienii* (Taxaceae)

**DOI:** 10.1186/s12864-021-07682-3

**Published:** 2021-05-26

**Authors:** Li Liu, Zhen Wang, Yingjuan Su, Ting Wang

**Affiliations:** 1 School of Life Sciences, Sun Yat-sen University, Guangzhou, Guangdong China; 2 Research Institute of Sun Yat-sen University in Shenzhen, Shenzhen, Guangdong China; 3 College of Life Sciences, South China Agricultural University, Guangzhou, Guangdong China

**Keywords:** *Pseudotaxus chienii*, Population transcriptome, SNP, Population structure, Genotype-environment association, Local adaptation

## Abstract

**Background:**

Elucidating the effects of geography and selection on genetic variation is critical for understanding the relative importance of adaptation in driving differentiation and identifying the environmental factors underlying its occurrence. Adaptive genetic variation is common in tree species, especially widely distributed long-lived species. *Pseudotaxus chienii* can occupy diverse habitats with environmental heterogeneity and thus provides an ideal material for investigating the process of population adaptive evolution. Here, we characterize genetic and expression variation patterns and investigate adaptive genetic variation in *P. chienii* populations.

**Results:**

We generated population transcriptome data and identified 13,545 single nucleotide polymorphisms (SNPs) in 5037 unigenes across 108 individuals from 10 populations. We observed lower nucleotide diversity (π = 0.000701) among the 10 populations than observed in other gymnosperms. Significant negative correlations between expression diversity and nucleotide diversity in eight populations suggest that when the species adapts to the surrounding environment, gene expression and nucleotide diversity have a reciprocal relationship. Genetic structure analyses indicated that each distribution region contains a distinct genetic group, with high genetic differentiation among them due to geographical isolation and local adaptation. We used *F*_ST_ outlier, redundancy analysis, and latent factor mixed model methods to detect molecular signatures of local adaptation. We identified 244 associations between 164 outlier SNPs and 17 environmental variables. The mean temperature of the coldest quarter, soil Fe and Cu contents, precipitation of the driest month, and altitude were identified as the most important determinants of adaptive genetic variation. Most candidate unigenes with outlier signatures were related to abiotic and biotic stress responses, and the monoterpenoid biosynthesis and ubiquitin-mediated proteolysis KEGG pathways were significantly enriched in certain populations and deserve further attention in other long-lived trees.

**Conclusions:**

Despite the strong population structure in *P. chienii*, genomic data revealed signatures of divergent selection associated with environmental variables. Our research provides SNPs, candidate unigenes, and biological pathways related to environmental variables to facilitate elucidation of the genetic variation in *P. chienii* in relation to environmental adaptation. Our study provides a promising tool for population genomic analyses and insights into the molecular basis of local adaptation.

**Supplementary Information:**

The online version contains supplementary material available at 10.1186/s12864-021-07682-3.

## Background

Dissecting the distribution of genetic variation across landscapes helps us to understand the ecological and evolutionary processes under climate change. The influence of natural selection on genetic variation and expression variation in natural populations has received increasing attention in studies on adaptive evolution and molecular ecology [1]. As species are forced to cope with environmental changes, it becomes increasingly important to understand how populations quickly adapt to diverse environments [2, 3]. Long-lived trees with a wide range of natural habitats often show clear adaptation to local environments [4]. Evidence for local adaptation can be detected if there is significant association with the environmental variables at some loci [5]. Individuals growing in different geographical areas will be subject to different selection pressures and therefore adapt to different local environmental conditions [4]. Genetic divergence may be caused by selection imposed by environmental pressures or the influence of genetic drift and limited gene flow when populations are partially isolated [6]. High levels of gene flow and continuous migration have homogenization effects, but natural selection is inferred to drive genetic divergence [7]. Describing spatial isolation and natural selection is essential for disentangling the processes that initiate genetic divergence, including the relative role of adaptation in driving differentiation and the number and identity of its potentially associated genetic targets.

With the development of sequencing technology, next-generation sequencing (NGS) has made it possible to obtain genome-wide scale sequence information across populations, greatly promoting the investigation of adaptive evolution and molecular ecology in nonmodel species [8]. Previous studies using anonymous markers (i.e., simple sequence repeat (SSR) and amplified fragment length polymorphism (AFLP)) were unable to assess the degree of linkage and the independence of loci, making them less reliable than other studies [9]. RNA sequencing (RNA-Seq) based on NGS can provide a more accurate estimate of the number of independent loci involved in adaptation and be used to detect potential candidate genes. RNA-Seq can be used to perform gene expression studies in species without genomic sequence information; thus, it is a very promising application in research on adaptation. Expression variation may occur before genetic variation and may be heritable [10, 11]; therefore, expression differences may reflect the early process of adaptive divergence at the population level [12]. In addition to identifying gene expression variations, RNA-Seq data can also allow the development of single-nucleotide polymorphisms (SNPs) on a large scale [13], which can capture potential sequence variations. These sequence variations and expression variations may be involved in the adaptation of a species to its natural habitat.

Transcriptome sequencing is a powerful tool that represents a cost-effective approach for examining genetic and expression patterns and investigating adaptive divergence at the levels of sequences, genes or biological metabolic pathways among natural populations in nonmodel organisms [14]. For example, Yan et al. (2017) [15] sequenced the transcriptomes of 78 *Miscanthus lutarioriparius* individuals from 10 populations and found genes related to photosynthetic processes and responses to environmental stimuli such as temperature and reactive oxygen species. Sun et al. (2020) [16] compared the transcriptomes of *Pinus yunnanensis* from high- and low-elevation sites and identified 103,608 high-quality SNPs and 321 outlier SNPs based on RNA-Seq to investigate adaptive genetic variation. The 321 outlier SNPs from 131 genes displayed significant divergence in terms of allelic frequency between high- and low-elevation populations and indicated that the flavonoid biosynthesis pathway may play a crucial role in the adaptation of *P. yunnanensis* to high-elevation environments. These studies provide insights into the patterns of genetic variation and gene expression in natural populations and aid in the exploration of loci involved in adaptation to diverse habitats.

The white-berry yew, *Pseudotaxus chienii* (W. C. Cheng) W. C. Cheng, is a threatened tertiary relict monotypic gymnosperm in the genus *Pseudotaxus* (Taxaceae) [17]. This species is a dioecious evergreen shrub or tree that grows in the subtropical mountains of China [17]. The distribution of *P. chienii* covers a relatively large geographical area with abundant environmental variation, in which includes mountain forests of northwestern Hunan, central Guangxi, southwestern Jiangxi, and southern Zhejiang [17]. Significant environmental heterogeneity has been found among most populations of *P. chienii* [18]. The wide range of natural habitats of *P. chienii* demonstrates its adaptability to various soils and growth conditions. Populations of *P. chienii* primarily grow in shallow and acidic soil, in rock crevices or on cliffs [19, 20]. *P. chienii* can adapt well to diverse habitats with environmental heterogeneity [20, 21] and thus provides an ideal material for investigating the process of population adaptive evolution. Morphological surveys of *P. chienii* in different geographical areas with different climatic conditions demonstrated that the width of the leaves gradually increases geographically from east to west [22], providing evidence for local adaptation of the plant phenotype. In plants, a large part of the phenotypic variation can be attributed to divergent selection imposed by environmental variables [23, 24]. Nevertheless, the main environmental variables that drive selection between natural populations are still unknown in most plants. The currently available data cannot provide a comprehensive understanding of the genetic status and adaptive divergence of *P. chienii* populations, and population genomic data from natural populations of this species are needed to solve these problems.

Adaptive genetic variation is common in tree species, especially widely distributed long-lived species [25]. Candidate loci/genes related to adaptive changes in different environments are increasingly included in investigations of adaptive divergence in trees [26]. In this study, we applied population transcriptome data to detect the genetic basis of local adaptation in *P. chienii* and determine which environmental variables are essential in driving population genetic differentiation. We detected 13,545 SNPs in 5037 unigenes across 10 populations using RNA-Seq. Population genetics and gene expression variation were explored. We integrated environmental and geographic information and used genetic loci to evaluate the impacts of environmental factors and geographic factors on genetic variation. The outlier SNPs associated with environmental variables and the candidate unigenes that contribute to local adaptation in *P. chienii* were also identified. The results of our study are expected to improve insights into evolutionary processes and local adaptation in *P. chienii*.

## Results

### De novo assembly and SNP calling

For 108 individuals, we obtained a total of 6336.45 Mbp raw reads with an average of 58.67 Mbp (Additional file 1). After the filtering process, 6258.14 Mbp clean reads representing 938.69 G bases were retained, with an average Q20 of 98.09%. Based on clean reads, 600,273 unigenes with a total of 426.75 Mbp nucleotide bases were assembled de novo. The mean N50 length and the mean length were 891 bp and 711 bp, respectively (Additional file 2). Of these unigenes, 230,731 (38.44%) were 301–500 bp, 172,167 (28.68%) were 501–1000 bp, 77,275 (12.87%) were 1–2 kb and 28,612 (4.77%) were more than 2 kb (Additional file 3). The final 600,273 unigenes from the 108 individuals were used as the reference sequences for *P. chienii*.

The clean reads of each individual were mapped to the reference sequences, and the mapping rates ranged from 66.48% in LMD_10 to 74.15% in DXG_7 (Additional file 4), indicating ideal mapping. We successfully identified 1,430,611 and 828,372 raw SNPs using GATK and SAMtools, respectively. After filtering steps, 84,974 and 57,196 SNPs were retained using GATK and SAMtools, respectively. To obtain high-quality SNPs, only SNPs identified by both SAMtools and GATK were retained. Overall, 13,545 SNPs from 5037 unigenes were identified across the 108 individuals from 10 populations.

### Genetic variation and population genetic structure

At the species level, the nucleotide diversity (π) of *P. chienii* was 0.000701. At the population level, LMD had the lowest π (0.000512), whereas LHS had the highest π (0.000723). The observed heterozygosity (*H*_O_) and expected heterozygosity (*H*_E_) of the 10 populations ranged from 0.383 (ZZB) to 0.493 (ZJJ) and from 0.356 (YSGY) to 0.422 (ZJJ), respectively (Table 1). Wright’s inbreeding coefficient (*F*_IS_) values were positive in all 10 populations. Regarding population differentiation, the *F*_ST_ value was highest between ZJJ and BJS (0.380), while MS and LMD had the lowest *F*_ST_ value (0.078) (Additional file 5). Moreover, the pairwise *F*_ST_ values of ZZB vs. BJS and LMD vs. SMJ were negative, implying that gene flow between these populations was common. We further tested the pairwise *F*_ST_ values among the four groups (see Methods section). The pairwise *F*_ST_ values among the four groups ranged from 0.216 (ZJ vs. JX) to 0.361 (HN vs. JX), suggesting that HN and JX had the greatest genetic distance (Additional file 6).

**Table 1:** Location information and genomic polymorphisms for 10 *Pseudotaxus chienii* populations

Population	Number of individuals	Location	Longitude (E)	Latitude (N)	Altitude (m)	π	***H***_**O**_	***H***_**E**_	***F***_**IS**_
BJS	12	Bijia Mountain, Jiangxi province	114°09′40″	26°30′31″	1293	0.000679	0.387	0.358	0.186
ZZB	12	Zizhuba, Jiangxi province	114°06′37″	26°29′25″	1297	0.000693	0.383	0.363	0.187
SQS	8	Sanqing Mountain, Jiangxi province	118°04′07″	28°54′03″	1343	0.000722	0.413	0.382	0.117
DXG	12	Daxiagu, Zhejiang province	119°10′14″	27°52′51″	1487	0.000702	0.387	0.364	0.159
LMD	11	Longmending, Zhejiang province	118°57′06″	28°43′42″	1049	0.000512	0.397	0.373	0.425
MS	12	Maoshan, Zhejiang province	118°58′23″	28°06′07″	1158	0.000721	0.407	0.372	0.111
SMJ	12	Shuimenjian, Zhejiang province	118°53′60″	28°43′37″	914	0.000598	0.388	0.365	0.320
LHS	12	Lianhua Mountain, Guangxi	110°06′53″	24°09′23″	1080	0.000723	0.431	0.365	0.112
YSGY	12	Yinshan Park, Guangxi	110°14′36″	24°09′60″	1182	0.000679	0.412	0.356	0.153
ZJJ	5	Zhangjiajie, Hunan province	110°28′53″	29°23′06″	1002	0.000721	0.493	0.422	0.261
Species level	108					0.000701	0.333	0.387	0.234

The parameters calculated the nucleotide diversity (π), observed heterozygosity (*H*_O_), expected heterozygosity (*H*_E_) and Wright’s inbreeding coefficient (*F*_IS_)

**Fig. 1:**
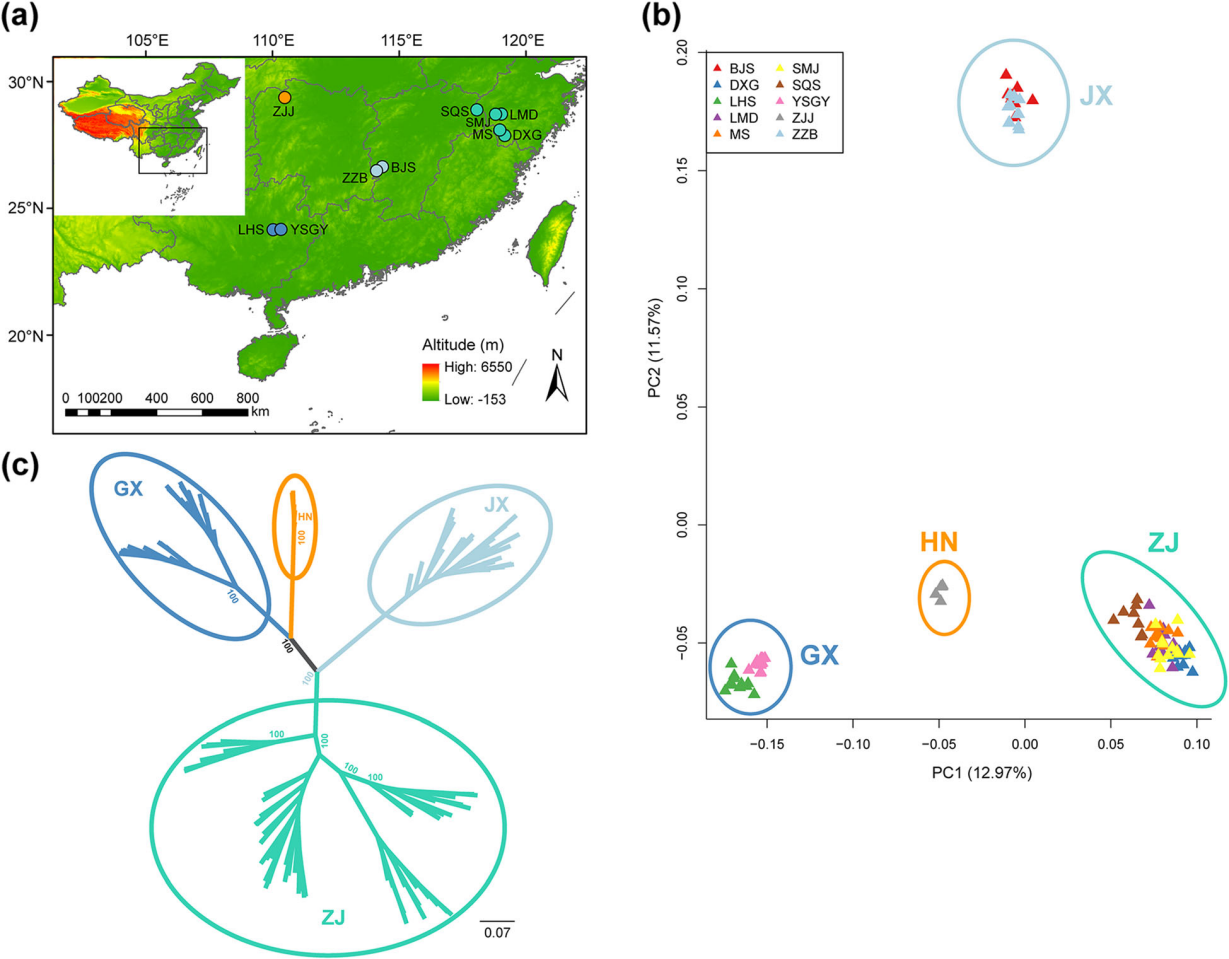
Geographical distributions and population structure of *Pseudotaxus chienii*. Colors denote the four main groups. **a** Sampling locations. Populations refer to those in Table 1. Colors denote the four main groups recovered from principal component analysis (PCA) and phylogenetic analysis. The map was downloaded from the National Geomatics Center of China (http://www.ngcc.cn/) and constructed using the ArcGIS ver. 10.4.1 (http://www.esri.com/software/arcgis/arcgis-for-desktop). **b** PCA of the 108 individuals based on the first two principal components. **c** A maximum likelihood (ML) tree based on SNPs from the transcriptome data

**Fig. 2:**
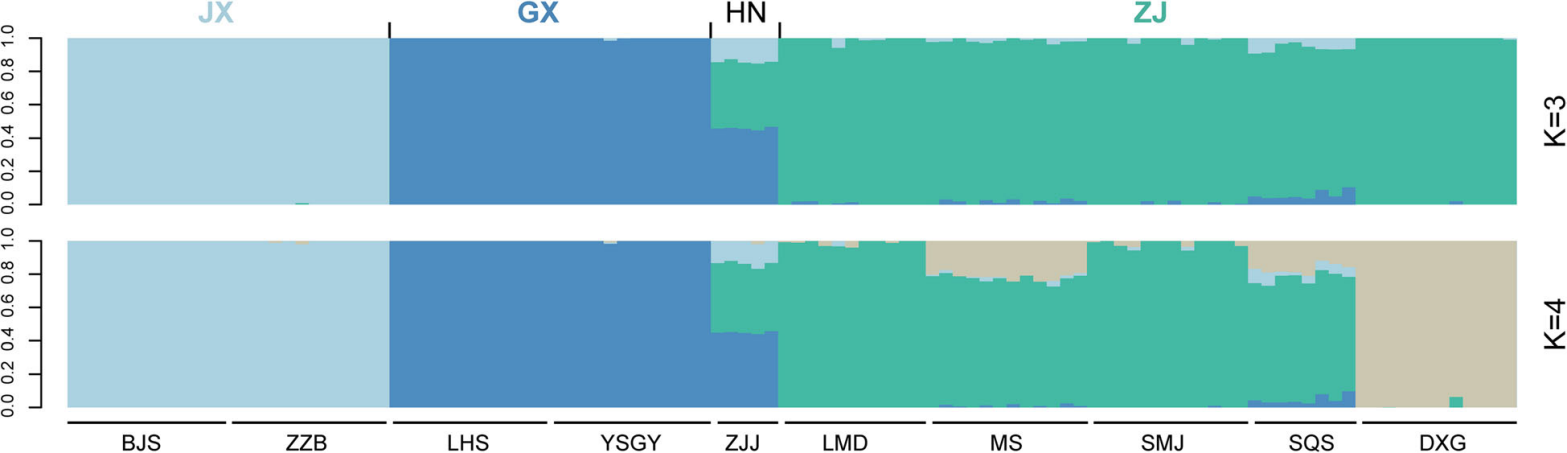
Admixture proportions indicating population genetic structure for each individual of *Pseudotaxus chienii*. The scenarios of *K* = 3 and *K* = 4 are shown. The cross-validation analysis showed that *K* = 4 was the optimal *K* value

Principal component analysis (PCA) unambiguously revealed four distinct genetic clusters. The first two principal components (PCs), which explained 12.97 and 11.57% of the total genetic variance, respectively, differentiated the four geographically distinct *P. chienii* groups: Zhejiang (ZJ: SQS, DXG, LMD, MS, and SMJ populations), Jiangxi (JX: BJS and ZZB populations), Guangxi (GX: LHS and YSGY populations), and Hunan (HN: ZJJ population) (Fig. 1b). These four groups corresponded almost entirely to separate geographic regions. To further explore the population genetic structure of *P. chienii*, genetic clustering of the 108 individuals was performed using ADMIXTURE, which also indicated that four genetic clusters (*K* = 4) was optimal with the lowest cross-validation error. With *K* = 4, individuals of the JX (BJS and ZZB populations), ZJ (LMD, MS, SMJ, and SQS populations), and GX (YSGY and LHS populations) groups clustered into three clusters, and the DXG population of the ZJ group was assigned to an independent cluster. The HN (ZJJ population) group contained a mixture of genetic components of the ZJ, JX and GX clusters (Fig. 2). Although *K* = 4 was the optimal *K* value, several other *K* values also showed biologically relevant patterns. When *K* = 3, DXG was clustered into the ZJ cluster, which was consistent with the geographical distribution of *P. chienii* and the PCA results.

A phylogeny based on 13,545 genome-wide SNPs showed three lineages, corresponding to ZJ, GX + HN, and JX (Fig. 1c). The JX lineage was at the most basal position, followed by GX + HN and then ZJ. Although the ADMIXTURE analyses showed that the HN group contained a mixture of genetic components of ZJ, JX and GX, phylogenetic analysis further confirmed that HN was closer to GX than JX or ZJ.

Analysis of molecular variance (AMOVA) of 13,545 SNPs revealed that 74.59% of the overall variation (*df* = 206; *p* < 0.0001) was distributed within populations and 25.41% among populations (*df* = 9; *p* < 0.0001) (Table 2). AMOVA found significant genetic differentiation among populations (*F*_ST_ = 0.254; *p* < 0.0001). The Mantel test detected a statistically significant correlation between pairwise *F*_ST_ and geographic distance among the 10 populations (*r* = 0.688, *p* = 0.001), indicating a significant pattern of isolation by distance (IBD). We also identified a significant pattern of isolation by environment (IBE) (*r* = 0.602, *p* = 0.001), and the level of correlation was similar to that of IBD.

**Table 2:** Analysis of molecular variance (AMOVA) of SNP data for *Pseudotaxus chienii*

Source of variance	Degrees of freedom (df)	Sum of squares	Variance components	Variance percentage (%)
Among populations	9	51,175.342	232.76646 Va	25.41
Within populations	206	140,720.070	683.10714 Vb	74.59
Total	215	191,895.412	915.87359	
Fixation index	*F*_ST_ = 0.254; *p* < 0.0001			

### Population gene expression variation

**Fig. 3:**
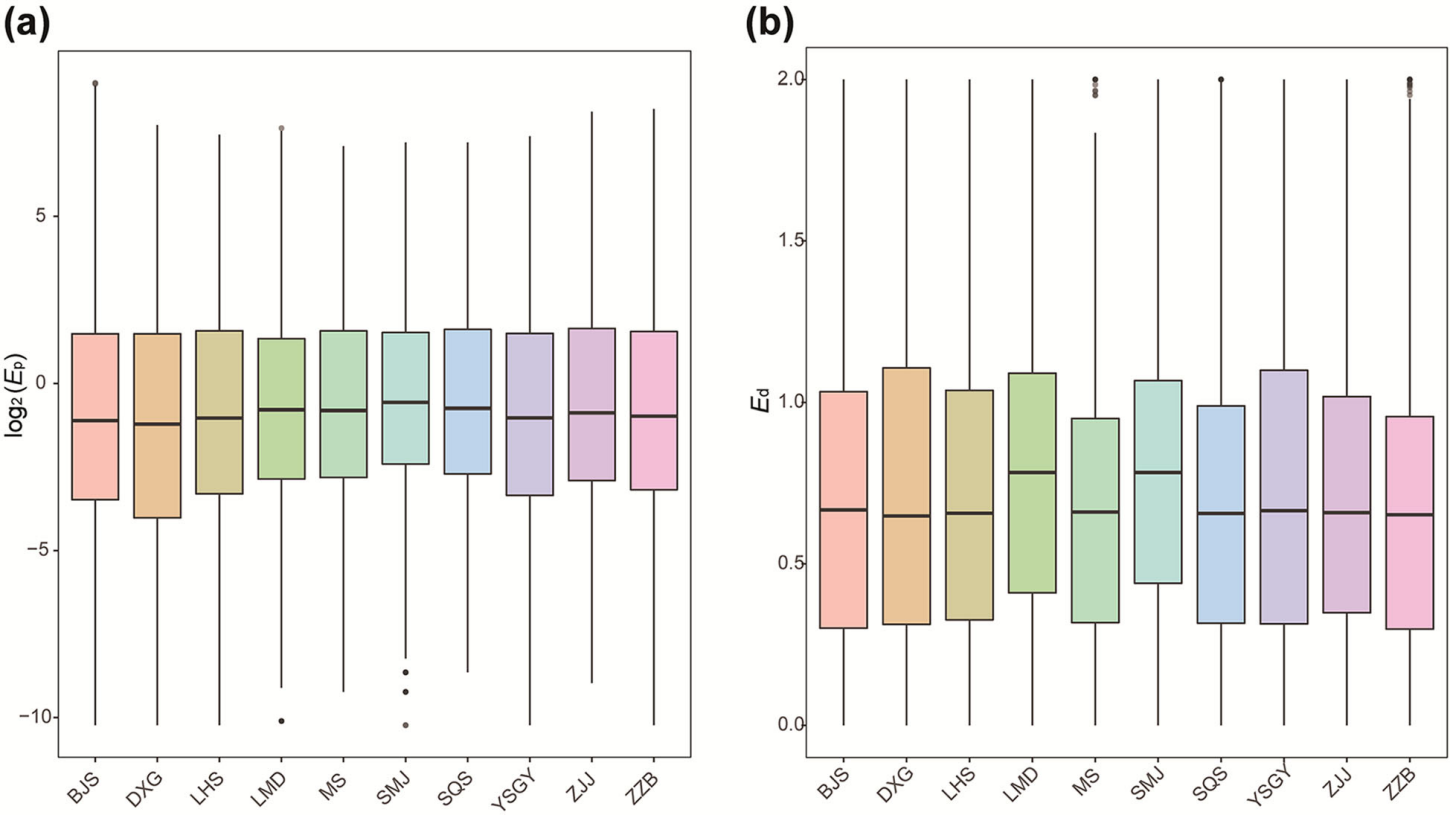
The quantiles of gene expression in 10 populations of *Pseudotaxus chienii*. **a** Population expression (*E*_p_). **b** Expression diversity (*E*_d_)

The population gene expression level (*E*_p_) and expression diversity (*E*_d_) were analyzed based on 108 *P. chienii* individuals from 10 populations. The distribution of *E*_p_ for 16,225 unigenes was right-skewed and peaked at expression level intervals of 0–10 (Additional file 7a). The quantiles of log_2_*E*_p_ in each population were similar (Fig. 3a). The average *E*_p_ values of the 10 populations ranged from 2.244 (SMJ) to 2.634 (ZJJ). *E*_d_ also showed a right-skewed distribution with a peak at 0.2–1.3 (Additional file 7b). The quantiles of *E*_d_ shifted down in LMD and SMJ (Fig. 3b). The average *E*_d_ values of the 10 populations ranged from 0.663 (MS) to 0.800 (LMD).

We further analyzed the relationship between *E*_d_ and π in each population. At the unigene level, the relationship between *E*_d_ and π in each population except BJS and MS showed a significant negative correlation (*r* = − 0.075 – − 0.032; *p* = 6.80 × 10^− 7^ – 0.031; Additional file 8). However, at the population level, there was no significant difference between the average *E*_p_ and π among the 10 populations (*r* = 0.39; *p* = 0.26; Additional file 9). Expression similarity (*E*_p_ similarity) was also not significantly correlated with genetic distance (*r* = − 0.07; *p* = 0.38; Additional file 10).

### Directional migration rates

**Fig. 4:**
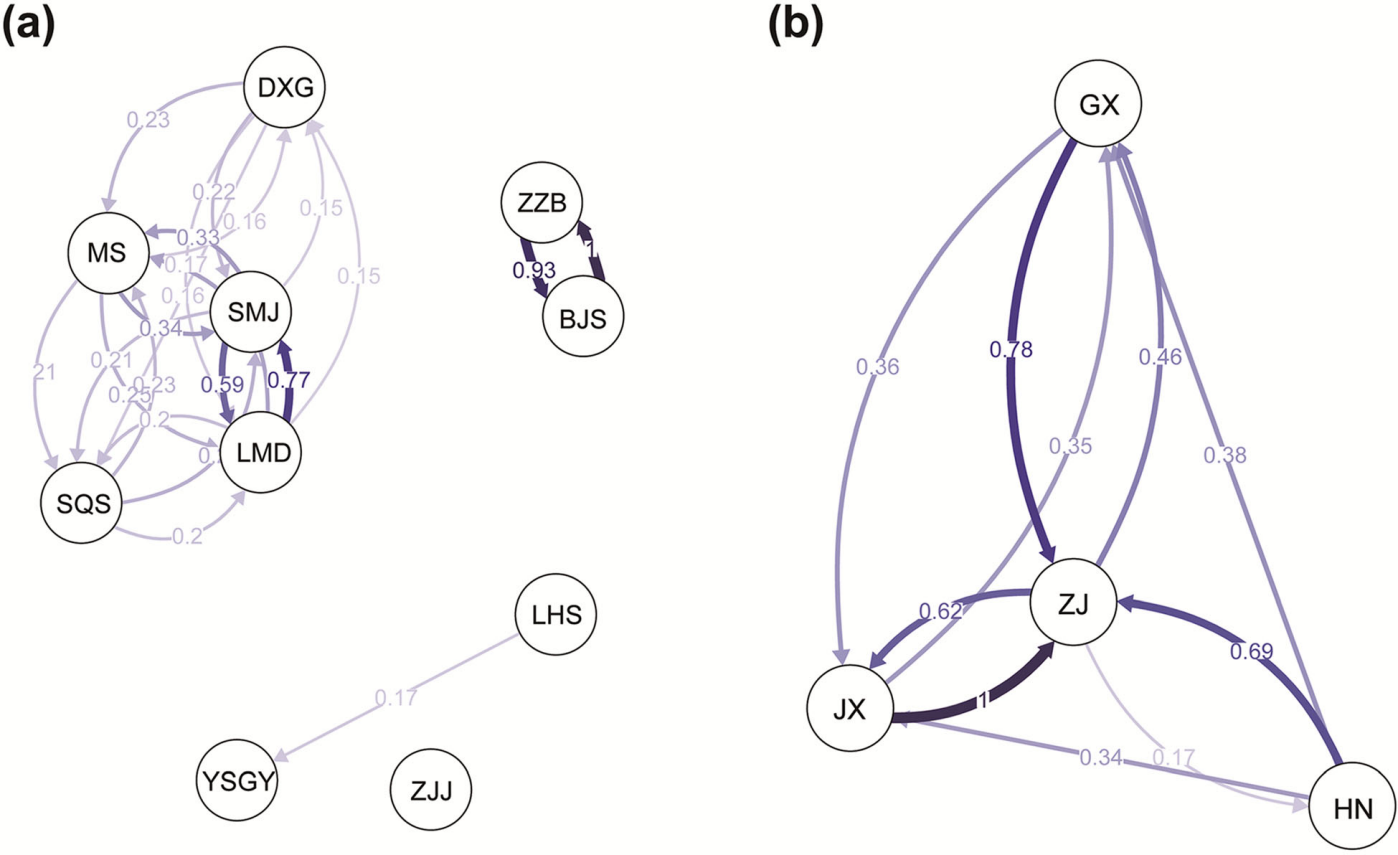
The bidirectional relative migration rates in *Pseudotaxus chienii* calculated using a putatively neutral dataset (12,566 SNPs). **a** Among 10 populations. **b** Among the four groups

The bidirectional relative migration rates (*m*_R_) among the 10 populations/four groups were similar across three measures (Jost’s *D*, *G*_ST_, and *N*m) of genetic differentiation; therefore, we describe the result based only on the *N*m (Fig. 4). Among the 10 populations, high relative migration rates were observed in both directions between BJS and ZZB (*m*_R_ > 0.90) and from LMD to SMJ (*m*_R_ = 0.77). The relative migration rates between LHS and YSGY (*m*_R_ = 0.17 for LHS to YSGY; *m*_R_ = 0.11 for YSGY to LHS) were lower than the migration rates between most populations in the ZJ group (SQS, DXG, LMD, MS, and SMJ) (Fig. 4a). Among the four groups, the highest relative migration rates (*m*_R_ = 1 for JX to ZJ) were observed. High relative migration rates were also observed from GX to ZJ (*m*_R_ = 0.78), from HN to ZJ (*m*_R_ = 0.69), and from ZJ to JX (*m*_R_ = 0.62) (Fig. 4b). Additionally, the relative migration rates between HN and ZJ were higher than those between HN and GX, despite the closer geographic proximity of HN and GX.

### Ecological niche differences among populations of *P. chienii*

**Fig. 5:**
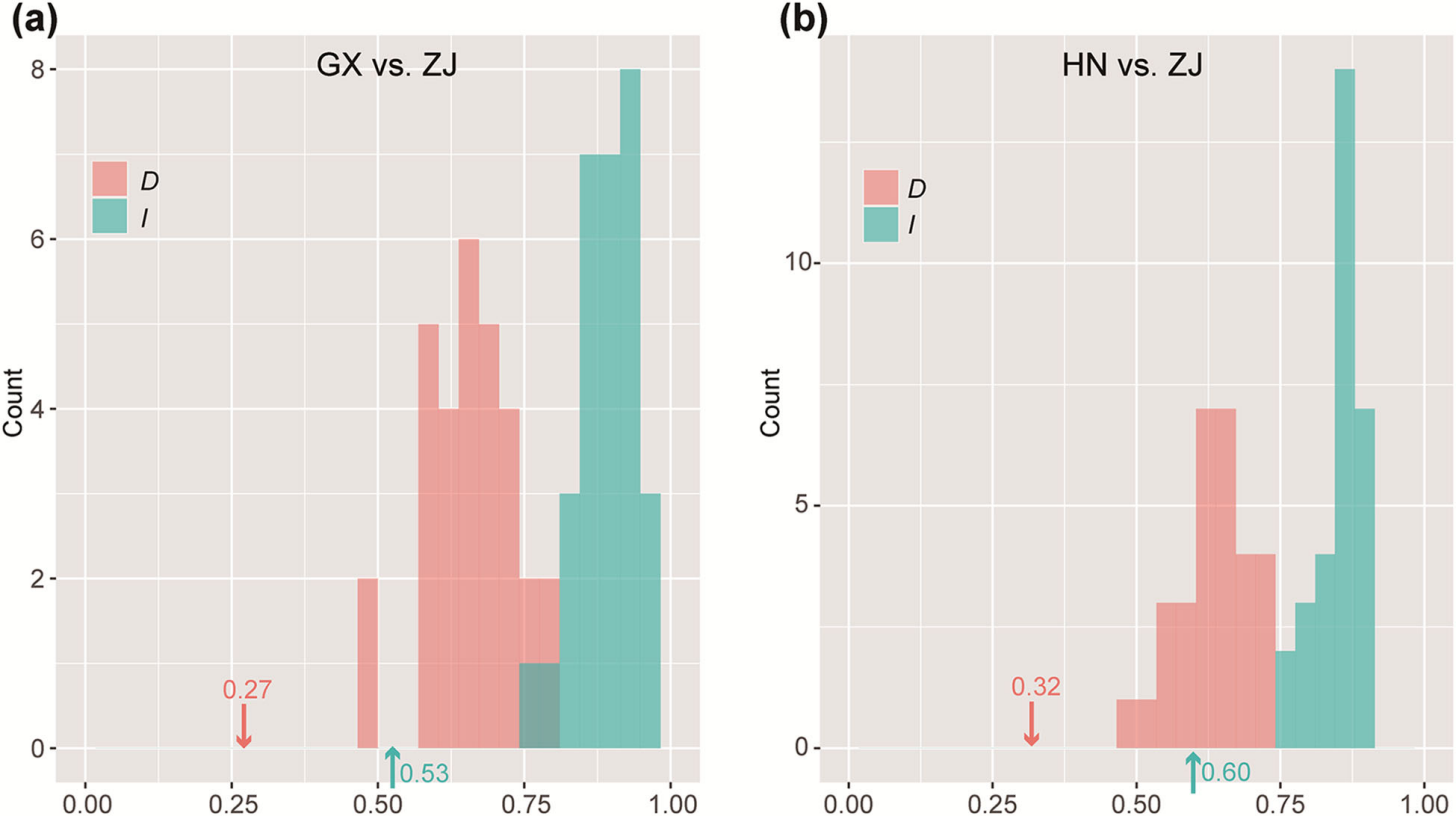
The niche differences between pairs of the four groups obtained using the niche overlap tool. The bars indicate the null distributions of Schoener’s *D* and the standardized Hellinger distance (*I*). Arrows indicate values of *D* and *I* in maxent runs. **a** GX vs. ZJ. **b** HN vs. ZJ

Ecological niche modelings were constructed for the four groups of *P. chienii* to predict their current, past and future potential distributions. All Maxent models for the four *P. chienii* groups had high predictive performance, with area under the receiver operating characteristic curve (AUC) values of 0.955 for the GX group, 0.955 for the HN group, 0.982 for the JX group, and 0.998 for the ZJ group. The mean temperature of the coldest quarter (64.87%), precipitation seasonality (CV) (73.24%), precipitation of the driest month (46.56%), and precipitation of the driest month (28.45%) made the largest independent contributions to GX, HN, JX, and ZJ, respectively (Additional file 11). The observed measures of Schoener’s *D* and standardized Hellinger distance (*I*) produced by Maxent runs were lower than the critical values of null distributions for GX vs. ZJ and HN vs. ZJ, indicating high niche differentiation between ZJ and both GX and HN (Fig. 5). However, the observed measures of *D* and *I* fell into the range of null distributions for the remaining four combinations; thus, few niche differences existed in these four combinations.

Under the current climate, the predicted distribution of *P. chienii* is basically consistent with the actual distribution of each group, although there are a few predicted areas where the species is not found, such as Taiwan. Under the interglacial (LIG) climate, JX, GX, and HN showed considerable contraction in suitable habitats, while clear range expansions were observed for the ZJ group. For the last glacial maximum (LGM) model, clear expansions in suitable habitats were predicted for all groups. The future distribution models showed a loss of suitable habitats for ZJ and JX relative to the current distribution, while the predicted current and future distributions were nearly identical for GX and HN (Additional file 12).

### Identification of outlier SNPs and unigene annotation

**Fig. 6:**
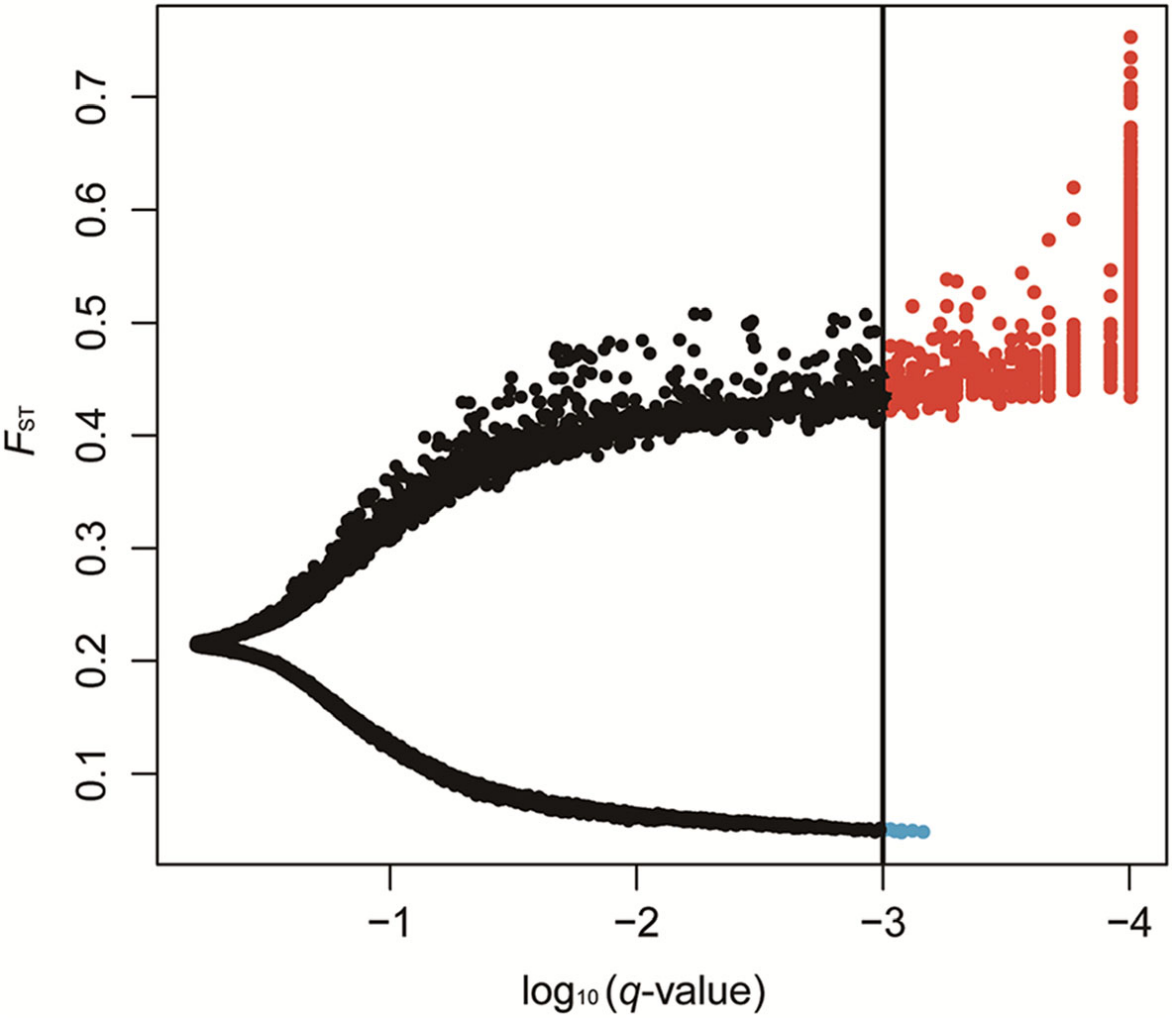
The scatter plot from Bayesian outlier analysis of SNPs, where SNPs with a *q*-value lower than 0.001 were considered outlier SNPs. The vertical black line indicates the cut-off with a *q*-value = 0.001; the red circles represent the outlier SNPs with positive α values; the blue circles represent the outlier SNPs with negative α values

We identified 979 outlier SNPs using BayeScan software with a 0.001 *q*-value threshold (Fig. 6), including 972 SNPs with diversifying selection and seven SNPs with purifying/balancing selection. The 972 outlier SNPs could be under divergent selection, revealing evidence of adaptive differentiation among the 10 populations. The *F*_ST_ estimated in BayeScan ranged from 0.047 to 0.753, with an average value of 0.224. Approximately 80% of the SNPs (10,980 of 13,545; 81.06%) showed *F*_ST_ < 0.25, while the *F*_ST_ values for outlier SNPs were high, with an average value of 0.503, suggesting that the 10 populations were indeed differentiated at outlier SNPs. These 979 outlier SNPs resided in 642 unigenes, of which 431 and 402 were annotated in the Pfam and SwissProt protein databases, respectively. Gene ontology (GO) terms were used to functionally classify the 642 unigenes, which were classified into three main categories: 337 unigenes in “biological process”, 381 unigenes in “molecular function”, and 216 unigenes in “cellular component” (Additional file 13). The top 15 GO terms of the three main categories identified for these unigenes are shown in Additional file 14. The GO enrichment analysis of 642 unigenes showed that “translation regulator activity” (GO:0045182) and “protein binding” (GO:0005515) were significantly enriched (*q*-values < 0.05) (Additional file 15).

**Fig. 7:**
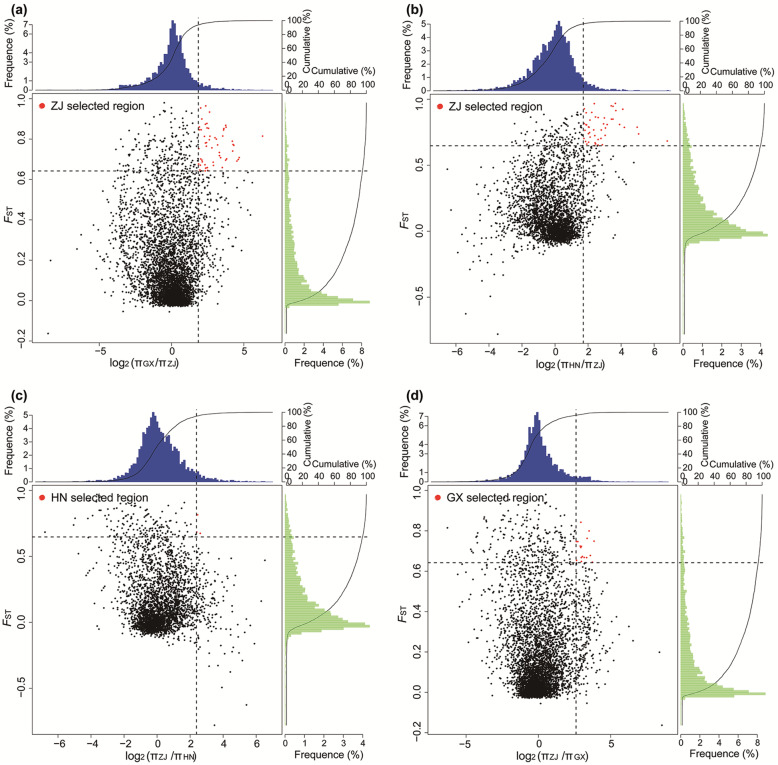
Selective sweep signals in *Pseudotaxus chienii*. The red points (corresponding to the top 5% of the log_2_(π ratio) distribution and the top 5% of the *F*_ST_ distribution) are genomic regions under selection in *P. chienii*. **a** Distribution of log_2_(π ratio) and *F*_ST_ values calculated between the Guangxi group (GX) and Zhejiang group (ZJ). **b** Distribution of log_2_(π ratio) and *F*_ST_ values calculated between the Hunan group (HN) and the ZJ group. **c** Distribution of log_2_(π ratio) and *F*_ST_ values calculated between the ZJ group and HN group. **d** Distribution of log_2_(π ratio) and *F*_ST_ values calculated between the ZJ group and GX group

Based on niche overlap analysis, the ecological differentiations of GX vs. ZJ and HN vs. ZJ were valid. Therefore, we further used selective sweep analysis to identify the unigenes underlying divergent adaptation in the ZJ, GX, and HN groups. Based on the top 5% of *F*_ST_ values and π ratio cutoffs (*F*_ST_ > 0.64 and 0.65 and log_2_(π ratio) > 1.85 and 1.70 for GX vs. ZJ and HN vs. ZJ, respectively; Fig. 7a, b), we identified 54 and 43 candidate unigenes involved in habitat adaptation in the ZJ group. These two unigene datasets contained 10 duplicated unigenes. Among the 87 candidate unigenes for habitat adaptation in the ZJ group, 56, 57 and 57 unigenes were annotated in the SwissProt, Pfam, and GO databases, respectively (Additional file 16). Kyoto Encyclopedia of Genes and Genomes (KEGG) enrichment analysis of these 87 candidate unigenes revealed one significantly overrepresented KEGG pathway with a *q*-value < 0.05: “monoterpenoid biosynthesis” (ko00902) (Additional file 17). Based on the top 5% of *F*_ST_ values and π ratio cutoffs (*F*_ST_ > 0.65 and log_2_(π ratio) > 2.38 for ZJ vs. HN; Fig. 7c), we identified three candidate unigenes involved in habitat adaptation in the HN group. The three candidate unigenes encode some proteins, including an AT-rich interactive domain-containing protein 2, an anaphase-promoting complex subunit 13, and the ETS transcription factor family, which is important for habitat adaptation in the HN group (Additional file 18). One significantly overrepresented KEGG pathway, “ubiquitin-mediated proteolysis” (ko04120), was identified (*q*-values < 0.05) (Additional file 19). Based on the top 5% of *F*_ST_ values and π ratio cutoffs (*F*_ST_ > 0.64 and log_2_(π ratio) > 2.61 for ZJ vs. GX; Fig. 7d), we identified 17 candidate unigenes involved in habitat adaptation in the GX group. Among the 17 candidate unigenes, 10, 9 and 9 unigenes were annotated in the SwissProt, Pfam, and GO databases, respectively (Additional file 20).

### Association of genomic variation with environmental variables

**Fig. 8:**
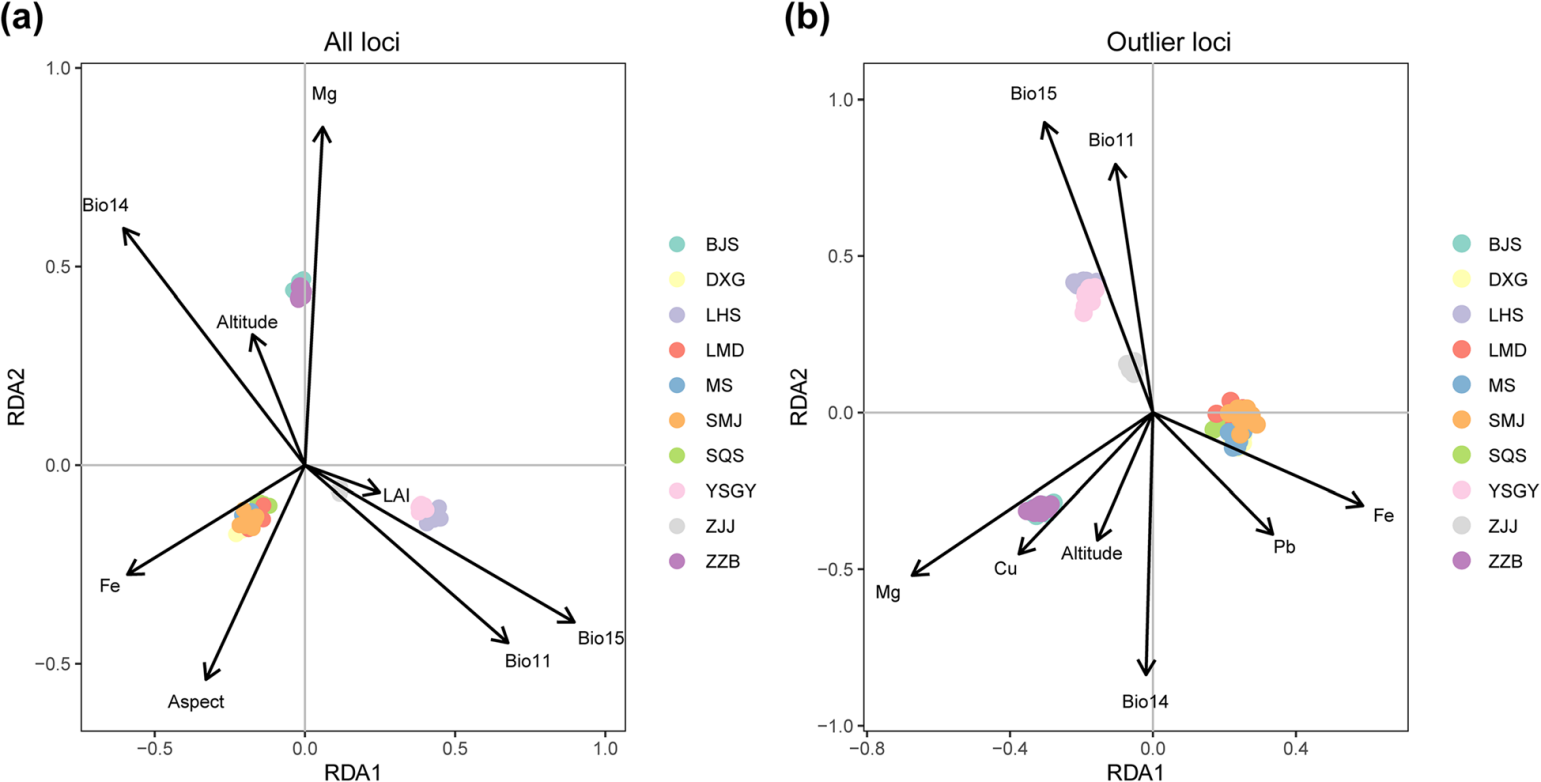
The results of redundancy analysis (RDA). **a** RDA1 and RDA2 axes of an RDA based on all loci. **b** RDA1 and RDA2 axes of an RDA based on outlier loci

We utilized the outlier test, redundancy analysis (RDA), and latent factor mixed models (LFMMs) to detect signatures of local adaptation among *P. chienii* populations and identify unigenes under selection. Forward selection of the environmental variables revealed two sets of eight environmental variables as significantly predictive of genetic variation for all loci and outlier loci (Additional file 21 and Fig. 8). The mean temperature of the coldest quarter, aspect, soil Fe content, precipitation of the driest month, and leaf area index were identified as the most important determinants of genetic variation for all loci, while the mean temperature of the coldest quarter, soil Fe content, soil Cu content, precipitation of the driest month, and altitude were the strongest determinants for outlier loci. The RDA axes were ordered by the amount of variance explained. Eight RDA axes (RDA1 to RDA8) explained 31.51% of the total genetic variance for all loci. The amount of explained variance increased to 64.06% when using only outlier loci as response variables. The permutation tests of the RDA models revealed *p*-values lower than 0.001 in these two analyses, thus confirming the high significance of the constrained variable effect.

Using all loci and outlier loci, we also carried out variation partitioning analysis to determine the relative contributions of environmental factors and geographic factors to the genetic variation. The models including all parameters ([a + b + c] in Table 3) showed a significant effect of these two factors (adjusted *R*^2^ = 0.6484, *p* = 0.001 for outlier loci; adjusted *R*^2^ = 0.3210, *p* = 0.001 for all loci). Environmental factors alone [a] (*F* = 4.0786, adjusted *R*^2^ = 0.0820, *p* = 0.001) and geographic factors alone [c] (*F* = 1.8585, adjusted *R*^2^ = 0.0059, *p* = 0.001) explained 8 and 1% of the variation at all loci, respectively; however, they explained 23% of the genetic variation when considered jointly [b] (adjusted *R*^2^ = 0.2331). Using outlier loci, pure environmental factors [a] explained 11% of the genetic variation (*F* = 11.815, adjusted *R*^2^ = 0.1130, *p* = 0.001), and pure geographic factors [c] explained 1% of the genetic variation (*F* = 3.1993, adjusted *R*^2^ = 0.0078, *p* = 0.001). Environmental factors and geographic factors together explained 53% of the genetic variation (adjusted *R*^2^ = 0.5276) (Table 3). In summary, the population divergence of *P. chienii* was strongly shaped by the joint effect of environmental factors and geographic factors, and environmental factors were more important than geography.

**Table 3:** Redundancy analyses (RDAs) to partition genetic variation in *Pseudotaxus chienii* into the environment (env.), geography (geo.), and their joint effects

	All loci			Outlier loci		
	Adjusted ***R***^**2**^	***F***	***p***-value	Adjusted ***R***^**2**^	***F***	***p***-value
RDA						
[a + b] gen. ~ env.	0.3151	7.1537	0.001	0.6406	24.836	0.001
[c + b] gen. ~ geo.	0.2390	7.7218	0.001	0.5354	21.552	0.001
Variation partitioning analysis						
[a] gen. ~ env. | geo.	0.0820	4.0786	0.001	0.1130	11.815	0.001
[c] gen. ~ geo. | env.	0.0059	1.8585	0.001	0.0078	3.1993	0.001
[b] gen. ~ geo. + env.	0.2331			0.5276		
[a + b + c] Total explained	0.3210	6.6205	0.001	0.6484	22.922	0.001
Total unexplained	0.6790			0.3516		

gen., SNP data matrix; env., environmental variables (all loci: eight environmental variables; outlier loci: eight environmental variables); geo., geographic variables (all loci: five dbMEM variables; outlier loci: six dbMEM variables)

To detect candidate outlier loci for local adaptation, we performed LFMM analyses that tested the correlations of single-locus–single-variable. We identified 244 associations between 164 outlier SNPs and 17 environmental variables (Additional file 22). Among the associations, 5 were related to temperature, 43 to precipitation, 65 to ecological factors, 43 to topographic variables, and 88 to soil variables. Only precipitation seasonality (CV) was not found to be associated with any outlier SNP. Of the other environmental variables, the fraction of absorbed photosynthetically active radiation was associated with the most outlier SNPs (36), followed by soil Cu content (29), soil Zn content (25), percent tree cover (21), aspect (18), and precipitation of the driest month (16).

These 164 outlier SNPs associated with environmental variables resided in 127 unigenes, of which 84, 93, and 35 were annotated in the SwissProt, Pfam, and KEGG databases, respectively (Additional file 23). Ninety-three unigenes were assigned to three main GO categories, including 71 unigenes in “biological process”, 83 unigenes in “molecular function”, and 46 unigenes in “cellular component”. For the biological process category, unigenes involved in “oxidation-reduction process” (GO:0055114), “transport” (GO:0006810), “ribosome biogenesis” (GO:0042254) and “signal transduction” (GO:0007165) were highly represented. In the molecular function category, the major GO terms were “protein binding” (GO:0005515), “ATP binding” (GO:0005524), and “zinc ion binding” (GO:0008270). The major terms for cellular component were “membrane” (GO:0016020), “integral component of membrane” (GO:0016021), and “nucleus” (GO:0005634).

## Discussion

The rapid development of sequencing technologies has provided powerful tools with which to investigate the genetic mechanisms in natural populations and new insights into the evolutionary and ecological processes underlying genetic differentiation and species adaptations [9, 27]. We used RNA-Seq, a commonly used NGS approach, to quantify the expression level of each unigene in each individual by mapping clean reads to reference sequences. SNP markers, third-generation molecular markers, have wide applicability, particularly SNP markers from transcriptome sequences, which can effectively reveal functional SNPs at the whole-genome level [28]. In this study, 13,545 high-quality SNPs were identified in 108 individuals of *P. chienii* based on transcriptome data to explore the driving mechanism of this species’ adaptations to its natural habitat. Our results show that very information in natural populations can be obtained from SNP markers of the *P. chienii* transcriptome.

### Population divergence and structure

Based on the transcriptome data from the populations, *P. chienii* had a lower nucleotide diversity (π = 0.000701) than other gymnosperms, such as *Cupressus chengiana* (π = 0.0077), *Cupressus duclouxiana* (π =0.0031), and *Cupressus gigantea* (π = 0.0029) [29, 30], suggesting that the sequences of protein-coding genes and functional elements of *P. chienii* captured by this method are highly conserved. Kou et al. (2020) [31] analyzed the genetic diversity of *P. chienii* populations using cpDNA sequences and nuclear loci and found similarly low nucleotide diversity (π = 0.0009) for cpDNA and more abundant diversity (π = 0.00265) for nuclear loci compared with those detected in this study. The difference may be due to the number of loci used in each analysis. Our estimates were based on 13,545 loci, whereas previous studies used only 14 nuclear loci. Therefore, we believe that the estimates of this study are more accurate than previous estimates. SNPs from RNA-Seq data are much more abundant DNA markers than other markers in plant genomes and have higher reproducibility, higher genotyping efficiency, and easier automation [32]. The measures of genetic diversity *H*_E_ and *H*_O_ were similar to the diversity calculated by expressed sequence tag-simple sequence repeat (EST-SSR) markers (*H*_E SNP_ = 0.387, *H*_O SNP_ = 0.333 vs. *H*_E EST-SSR_ = 0.370, *H*_O EST-SSR_ = 0.341) [18]. Furthermore, the ZJJ and LMD populations appeared to harbor relatively high genetic variation compared with that in other populations, suggesting that the ZJJ and LMD populations have undergone adaptation in response to natural selection. After the genetic admixture event (see below), the mixed population (ZJJ population) may experience its own unique genetic variation, resulting in higher genetic diversity. This phenomenon is common in long-lived gymnosperms, such as *C. chengiana* [29]. Positive *F*_IS_ values were observed in all sampled populations, suggesting significant heterozygote deficits. Heterozygote deficits are probably caused by several factors, including inbreeding, linkage disequilibrium, null alleles, recent admixture, and partial clonality [33, 34]. In our study, inbreeding and/or recent admixture seemed to be the most likely driver of the positive *F*_IS_ values, as investigations of *Taxus yunnanensis* (*F*_IS_ = 0.228) and *Taxus wallichiana* (*F*_IS_ = 0.290) showed prevalent inbreeding [35, 36].

The genetic differentiation levels among *P. chienii* populations/groups were generally high (*F*_ST_ > 0.15), except for a few pairs of populations in geographical proximity. The spatial context of natural selection and the balance between the strength of divergent selection and migration rates between populations/groups are of great significance to genetic differentiation [37, 38]. The high genetic differentiation levels may occur because most *P. chienii* grows in the understory; therefore, the biological characteristics of wind pollination may not help *P. chienii* spread pollen over a long distance. Additionally, the seed cone of *P. chienii* possesses a cup-like, fleshy, white aril [39] and mainly depends on biotic dispersal. Seeds with white arils are less attractive to birds and mammals than brightly colored seeds, such as those of *Taxus* species [40], which limits the spread of *P. chienii* seeds. From ecological and evolutionary perspectives, the species has been exposed to stressors or diverse environments, which has resulted in genetic differentiation and genetic heterogeneity. A few populations in geographical proximity showed relatively low genetic differentiation, possibly due to high migration rates and a high rate of effective pollen and seed dispersal. In this study, geographic distance and environmental distance were correlated with genetic differentiation (IBD: *r* = 0.688, *p* = 0.001; IBE: *r* = 0.602, *p* = 0.001), suggesting that IBD and IBE were important to the divergence among *P. chienii* populations. These results further revealed that the genetic differentiation among the *P. chienii* populations was mainly the result of geographical isolation and habitat heterogeneity.

Generally, a higher level of genetic differentiation indicates a stronger population structure. Genetic differentiation among populations with different geographic distributions was found in the ADMIXTURE and PCA analyses in our study. The ADMIXTURE results suggested the presence of three major genetic clusters and a smaller cluster (DXG) across the species’ natural range (Fig. 2), whereas three clusters were identified for two chloroplast regions and 14 nuclear loci [31]. Our results show that SNP markers based on transcriptome data are better able to detect fine-scale population structure than classic genetic markers. Here, ADMIXTURE analysis revealed that the ZJJ population was genetically admixed to the GX, ZJ and JX groups. Intuitively, this isolated population ZJJ had little chance of leaving the footprint of admixture introduced by other groups. However, considering that the HN group had experienced multiple expansions and contractions during the Quaternary climate oscillations [41], it was plausible that genetic admixture was established through gene flow between populations. Then, the specific habitat requirements of this group caused it to persist only in montane regions, and other low-altitude populations might extirpate due to local maladaptation, creating the geographically isolated population ZJJ. Overall, the transcriptome data based on high-throughput sequencing used here provide abundant markers that can contribute to the accurate description of admixture signals [42].

### Population gene expression variation

Gene expression variation among populations may be due to developmental, environmental, genetic or other biological effects, which are essential for adaptive evolution [11]. Understanding the patterns of genetic variation and gene expression in populations from different habitats can reveal the response of plants to different environments through variations in gene expression and/or allelic characteristics. In our data, we found significant negative correlations between expression diversity and nucleotide diversity in eight populations. This result suggests that when the species adapts to the surrounding environment, gene expression and nucleotide diversity have a reciprocal relationship. This phenomenon might be attributed to gene duplication events occurring in the *P. chienii* genome during the evolutionary process [43]. Compared with single-copy genes, duplicated genes usually significantly increase the diversity of gene expression, while genetic diversity remains relatively weak [44]. Higher gene expression diversity may have balanced the effects of lower genetic variation, thereby maintaining the stability of the phenotype under long-term natural selection in native habitats. We speculate that genetic variation and expression diversity both played a potential role in local adaptation.

### Evidence for local adaptation

Local adaptation to environmental variables is generally believed to play a major role in the diversification of species, but its contribution relative to those of other evolutionary forces is rarely quantified. Despite the strong population structure in *P. chienii*, analysis of genomic data revealed signatures of divergent selection associated with environmental variables. The *F*_ST_ outlier approach, RDA, the LFMM method, and selective sweep analysis were used to detect signatures of local adaptation among *P. chienii* populations and identify unigenes under selection. Environmental association studies have been more robust in identifying loci under selection and can also provide context for selection forces [45]. Testing for IBD and IBE with Mantel test revealed that environmental and geographic distances were important to the divergence among *P. chienii* populations. We further applied RDA to dissect the individual roles of environmental factors and geographic factors and their confounding effect on genetic variation. Our RDA showed that population divergence in *P. chienii* was strongly shaped by the joint effect of environmental factors and geographic factors, and environmental factors were more important than geography, a pattern consistent with local adaptation. Environmental differences among populations may constitute key factors maintaining genetic differentiation despite high relative migration rates between local populations. It follows that environmental differences among populations are closely related to the maintenance of genetic variation and that local adaptation may be the main driving force of these patterns. However, a large part of the variation remains unexplained. This may be due to several factors that cannot be fully resolved. First, although we included a large number of environmental variables in our study, many other unmeasured ecological forces may also play a role. Second, other evolutionary forces that maintain local genetic diversity, such as balancing selection, may weaken the signal of locus-environment associations [46]. Third, the multivariate environmental association approach models only linear associations, so nonlinear statistical relationships will not be captured.

In this study, we found evidence for local adaptation signals to genetic variation associated with environmental variables. We detected 164 SNPs residing in 127 unigenes as candidate targets of adaptive importance. The GO annotation analysis of 127 unigenes showed that the majority of the unigenes were related to abiotic and biotic stress responses, which is of particular interest for future population genomic research. In the biological process category, oxidation-reduction process, signal transduction, and protein phosphorylation were the most represented GO terms, which is consistent with the findings of adaptability studies in conifers and other plants. These three biological processes were shown to be involved in the drought response in Masson pine (*Pinus massoniana*) [47]. The oxidation-reduction process may have contributed to cold resistance in the mature leaves of tea plants (*Camellia sinensis*) [48] and adaptation to hypoxia, extreme temperatures, and high ultraviolet (UV) radiation in *Kandelia obovata* [49]. Protein phosphorylation is involved in activating cold acclimation [50]. Signal transduction connects sensing mechanisms with genetic responses, which is important for sensitivity to environmental stresses and promotes effective downstream processes in response to these environmental stresses [51]. Within the molecular function category, the term binding, including protein binding, ATP binding, and DNA binding, was generally related to the environmental stress response. The GO term binding was shown to be enriched in upregulated open reading frames (ORFs) associated with the cold response in Chinese yew (*Taxus chinensis*) [52]. Additionally, lines of evidence support that the membrane plays a key role in abiotic stress and plant defense. The membrane can directly or indirectly sense stress through physical properties to initiate signal transduction pathways [53, 54]. Previous studies revealed significant changes in plasma membrane function in response to cold stress in *T. chinensis* [52]. Although the annotation analysis suggested functional importance for most candidate unigenes, the unannotated unigenes are still promising candidates for future study, as they may be related to adaptive genes or genes of unknown importance.

The allele frequency of SNPs changes under selection pressure, thereby rapidly maximizing adaptability in different environments. Niche differentiation was detected for GX vs. ZJ and HN vs. ZJ; thus, we further used selective sweep analysis to identify the unigenes underlying divergent adaptation in the ZJ, GX, and HN groups. We found 87 candidate unigenes for habitat adaptation in the ZJ group. The KEGG pathway of monoterpenoid biosynthesis was significantly enriched (*q*-values < 0.05) for 87 candidate unigenes. Terpenoids play an important role in abiotic and biotic stresses and are involved in the defense against pathogens and herbivore attack in conifers [55]. Drought can increase the concentration of monoterpenoids in conifers, such as *Picea abies* and *Pinus halepensis* [56, 57]. Monoterpenoid biosynthesis may play an important role in the local adaptation of *P. chienii* in the ZJ group. Ubiquitin-mediated proteolysis was significantly enriched (*q*-values < 0.05) for candidate unigenes for habitat adaptation in the HN group. Ubiquitin-mediated proteolysis impacts many aspects of plant growth and development, including plant hormone signal transduction, reproduction, and abiotic stress responses [58]. Some genes involved in ubiquitin-mediated proteolysis show signs of positive selection [59]. The identified candidate unigenes for habitat adaptation in the GX group are directly or indirectly related to biotic or abiotic stress responses. For instance, Cluster-242,496.98097, encoding a 70 kDa heat shock protein (HSP70), is associated with pathogen and disease resistance and plant responses to high temperatures [60]. These results suggest that these pathways and candidate unigenes may play an important role in local adaptation in the GX, ZJ, and HN groups of *P. chienii*.

Detecting associations between SNPs and environmental variables helps us recognize the ecological, bioclimatic, and topographic variables that influence genetic variation. We found that the fraction of absorbed photosynthetically active radiation, soil Cu content, soil Zn content, percent tree cover, aspect, and precipitation of the driest month were associated with the most outlier SNPs when using the LFMM method (Additional file 22), suggesting their importance in shaping the genetic variation underlying *P. chienii* adaptability. The forward selection performed as part of RDA showed that the mean temperature of the coldest quarter, soil Fe content, soil Cu content, precipitation of the driest month, and altitude were important determinants of outlier genetic variation. These variables may be selective factors driving the local adaptation of *P. chienii*. Aspect, a topographic factor, is a key predictor of differences in forest radiation exposure and has a strong influence on the microclimate [61]. This factor has been found to be related to genetic variation within and among *Salix* species [62]. Both the LFMM method and RDA revealed a strong signal of divergent selection in relation to precipitation-related variables. Zhang et al. (2020) [41] highlighted the importance of precipitation of the driest month in shaping the species distribution of *P. chienii*. Precipitation can directly affect soil water content, thus affecting the absorption and transport of plant water and nutrients. A decrease in precipitation of the driest month is expected over the coming decades (current with a mean of 45.9 mm; 2050 with a mean of 33.2 mm), particularly in the distribution range of the JX group of *P. chienii*. In the case of rapid climate change, if *P. chienii* populations cannot adapt to increasing drought, ecological benefits will be greatly damaged. According to a global assessment, due to droughts, high temperatures, and insect outbreaks under climate change, the mortality rate of forest trees may increase [63]. Selecting and planting genotypes adapted to climate change is of great significance to the protection of the endangered species *P. chienii*. The identified candidate unigenes are directly or indirectly related to biotic or abiotic stress responses. For instance, Cluster-242,496.114750, encoding an LRR receptor-like serine/threonine-protein kinase, is associated with pathogen and disease resistance [64]. Candidate unigenes encoding one methyltransferase, four ubiquitin, and one auxin-responsive protein were detected (Additional file 23). They were associated with multiple environmental variables, including leaf area index, percent tree cover, fraction of absorbed photosynthetically active radiation, altitude, precipitation of the driest month, and soil Mg, Zn, Cu, and Mn contents. These proteins have been reported to be related to loblolly pine adaptability [65]. These detected unigenes with functional annotations provide strong support for adaptive variation in *P. chienii*. Future climate trends in the distribution range of *P. chienii*, including increased temperature and decreased precipitation, will pose challenges to *P. chienii* in terms of its environmental adaptation. Our research provides SNPs and candidate unigenes related to environmental variables to facilitate elucidation of the genetic variation and structure of *P. chienii* in relation to environmental adaptation.

## Conclusions

We identified 13,545 SNPs to determine genetic and expression variation patterns and local adaptation across 10 populations of *P. chienii*. Gene expression and nucleotide diversity had a reciprocal relationship when *P. chienii* adapted to the surrounding environment. Despite the strong population structure in *P. chienii*, genomic data revealed signatures of divergent selection associated with environmental variables. We identified 244 associations between 164 outlier SNPs and 17 environmental variables. The mean temperature of the coldest quarter, soil Fe and Cu contents, precipitation of the driest month, and altitude were identified as the most important determinants of adaptive genetic variation. Most candidate unigenes with outlier signatures were related to abiotic and biotic stress responses. The results of our study are expected to improve insights into evolutionary processes and local adaptation in *P. chienii*.

## Methods

### Sample collection and RNA isolation

Our sampling work complies with the laws of the People’s Republic of China and has a permission letter from Sun Yat-sen University. Voucher specimens were identified by Prof. Zien Zhao (Wuhan Botanical Garden, Chinese Academy of Sciences, Wuhan, Hubei, China) and kept at the Herbarium of Sun Yat-sen University (No: ds-2018-1001–ds-2018-1010).

Fresh and mature needles of 108 individuals from 10 populations of *P. chienii* were collected from first-year branches across the distribution range in China in May 2018 (Fig. 1a). A global positioning system (GPS) was used to record the geographic coordinates of the sampling locations. Plants were sampled at intervals of 20 m in each population. All samples were washed with purified water, cut into pieces and then immediately stored in RNAfixer (BioTeke, Shanghai, China). The RNAfixer with samples was stored in a − 20 °C freezer until further use.

Total RNA extraction of each individual was performed using the RNAprep Pure Plant Kit following the protocol of the manufacturer (Tiangen, Beijing, China). The purity and integrity of the extracted RNA were detected using a NanoDrop spectrophotometer (Thermo Scientific, DE, USA) and an Agilent 2100 Bioanalyzer (Agilent Technologies, CA, USA), respectively. An RNA integrity number > 6.0 was required for cDNA synthesis and library construction. One microgram of RNA from each individual was used for cDNA library preparation.

### Library construction, sequencing, and assembly

The Illumina library for each individual was constructed using the NEBNext Ultra™ RNA Library Prep Kit (NEB, MA, USA) following the manufacturer’s protocol. Poly(A) mRNA was enriched from total RNA using oligo (dT) magnetic beads. Then, the poly(A) mRNA was fragmented into small pieces using divalent cations under elevated temperature in NEBNext First Strand Synthesis Reaction Buffer (5×). The RNA fragments were reverse transcribed into first-strand cDNA using random hexamer primers and M-MuLV Reverse Transcriptase (RNase H-). Subsequently, second-strand cDNA was synthesized using dNTPs, DNA polymerase I, and RNase H. The purified double-strand cDNA was end-repaired and A-tailed, and then Illumina paired-end adapters were ligated. The library fragments were purified using AMPure XP beads (Beckman Coulter, Beverly, USA) to select cDNA fragments with lengths of 250–300 bp. Then, PCR was performed with Phusion High-Fidelity DNA polymerase, universal PCR primer and index (X) primer. After size selection and PCR amplification, qualified cDNA libraries were sequenced on the Illumina NovaSeq platform, generating paired-end reads with a length of 150 bp.

Raw Illumina RNA-Seq reads were filtered via in-house Perl scripts. Clean reads were obtained by removing the reads with more than 10% ambiguous bases (“N”), adapter reads, and Qphred scores ≤20 bases with more than 50% from the raw reads. Finally, clean reads of 108 individuals were de novo assembled to obtain the final unigenes with Trinity v.2.4.0 [66]. The final high-quality unigenes of 108 individuals served as the reference sequences for estimating genetic and expression variation among the 10 populations of *P. chienii*.

### Read mapping and SNP calling

Clean reads for each individual were mapped to the reference sequences of *P. chienii* using Bowtie 2 (http://bowtie-bio.sourceforge.net/bowtie2/index.shtml) with default parameters. Duplicate reads were excluded by Picard tools ver. 1.96 (http://broadinstitute.github.io/picard/); then, the reads were sorted and indexed in BAM format using SAMtools ver. 1.4 with default settings [67]. mpileup of SAMtools was used to analyze the alignment results of the reference sequence base sites with the parameters -q 1 -C 50 -t SP -t DP -m 2 -F 0.002. SNP calling was conducted with BCFtools of SAMtools using the following parameters: -Q 20 -d 1 -D 1000 -a 2 -w 3 -W 10. To ensure the accuracy of SNP identification, we also used GATK ver. 3.7 [68] to identify the SNPs with the parameters FS < 30.0, QD > 2.0, and DP > 10. All monomorphic SNPs were removed. Only SNPs identified by both SAMtools and GATK were retained. To minimize false-positive SNPs and obtain high-quality SNPs, we filtered the SNP loci using the criteria depth > 2, call rate > 0.5, and minor allele frequency (MAF) > 0.05 and kept only biallelic SNPs. For consistency with the gene expression data, we removed SNPs contained in unigenes with extreme fragments per kilobase of transcript per million mapped reads (FPKM) values (see below).

### Genetic variation and population genetic structure based on SNP data

Based on the SNP data, the nucleotide diversity (π) per site was calculated using VCFtools ver. 0.1.11 (https://vcftools.github.io/index.html) with nonoverlapping 1000-bp genomic windows. Pairwise genetic differentiation (*F*_ST_), observed heterozygosity (*H*_O_), and expected heterozygosity (*H*_E_) were calculated using Arlequin ver. 3.5.1.2 [69], and the significance of *F*_ST_ was determined using 1000 permutations. Wright’s inbreeding coefficient (*F*_IS_) was estimated for each population using the basic.stats function in the R package ‘hierfstat’ [70].

The population genetic structure of the 108 individuals was examined in ADMIXTURE ver. 1.23 [71] using the maximum-likelihood method to identify evolutionary clusters. The number of genetic clusters (*K*) was set from 2 to 10. Cross-validation error (CV error) was used to determine the most likely number of clusters. The lowest CV error indicated the optimum *K* value. GCTA ver. 1.93.2 software [72] was used to perform PCA on the *P. chienii* individuals. The first two components were plotted for *P. chienii* to explore its genetic structure.

AMOVA was used to assess the extent of genetic structure within and among populations as implemented in Arlequin, and the significance was determined for 1000 permutations.

The best model of nucleotide substitution was identified with PhyML 3.0 [73, 74] using Akaike’s information criterion (AIC), and GTRGAMMAI was the best-fitting model. A maximum likelihood (ML) tree was constructed using RAxML ver. 8.2.4 [75] with 1000 bootstrap replicates.

### Gene expression variation and population differentiation based on FPKM values

The clean reads of each individual were mapped to the reference sequences of *P. chienii* using Bowtie 2 of the RSEM software (http://bowtie-bio.sourceforge.net/Bowtie2/index.shtml). The readcount values of each unigene for 108 individuals were obtained. Considering the influence of the gene length and sequence depth on the fragments, all readcounts were normalized to the FPKM values. The FPKM values were calculated using the formula below: FPKM = (10^9^ × C)/(N × L), where C is the number of fragments mapped to the transcript, N is the total number of mappable reads, and L is the length of the transcript [76]. The expression level of each unigene in 108 individuals was determined by calculating the FPKM value. To filter the extremely large FPKM values, 1 was added to the FPKM value of each unigene, and then log_2_ transformation was performed. Quartile analysis was used to filter out values greater than 1.5 times the interquartile range [77, 78]. In total, 16,225 unigenes with at least half of the individuals with log_2_-transformed FPKM values larger than 0 were retained.

Gene expression variation in populations was evaluated using Xu et al.’s (2015) [79] method. The population gene expression level (*E*_p_) was evaluated as the average FPKM value of the individuals from the population. Expression diversity (*E*_d_) was calculated as the gene expression variation in the population. The formulas for *E*_p_ and *E*_d_ were based on Xu et al.’s (2015) method. To estimate the gene expression level relationship among populations (*E*_p_ similarity), we calculated Pearson’s correlation coefficients (*r*) based on the average correlation coefficients of 108 individuals. The significance of the relationship between genetic distance and *E*_p_ similarity in populations was tested with a Mantel test using 1000 random permutations. To detect the relationship between nucleotide diversity and expression diversity within populations, Pearson’s correlation coefficients (*r*) of π and *E*_d_ for 16,225 unigenes in each population were calculated. Additionally, Pearson’s correlation analysis was performed between π and *E*_p_ among populations. The ‘HMISC’ package in R was used for Pearson’s correlation analysis, and the significance was determined for 1000 permutations [80].

### Directional migration rates

We pooled the populations into four groups (Jiangxi group, JX: BJS and ZZB; Guangxi group, GX: LHS and YSGY; Hunan group, HN: ZJJ; and Zhejiang group, ZJ: SQS, SMJ, MS, LMD, and DXG) based on the results from the PCA, phylogenetic tree, and geographical distribution. To assess directional relative migration rates among the 10 populations/four groups of *P. chienii*, a putatively neutral dataset (12,566 SNPs) was performed with the divMigrate function in the R package ‘diveRsity’ [81] based on three measures of genetic differentiation (Jost’s *D*, *G*_ST_, and *N*m). This approach is a relative bidirectional measure of gene flow using all available genetic differentiation measures to evaluate the consistency of estimates among measures. The confidence interval (95%) of the relative migration rates was calculated with 1000 bootstrap iterations.

### Environmental variables

The environmental variables include 19 bioclimatic, 25 ecological, and three topographic variables. Bioclimatic data (averaging over the period 1950–2000) comprising 19 bioclimatic variables at a spatial resolution of 2.5 arc-mins were obtained from the WorldClim database (http://www.worldclim.org). The ecological variables comprised five ecological factors and 20 soil variables. The five ecological factors included the normalized difference vegetation index, percent tree cover, leaf area index, enhanced vegetation index, and fraction of absorbed photosynthetically active radiation, which were obtained from the Land Process Distributed Active Archive Center (http://lpdaac.usgs.gov, recorded in 2001–2018) based on the moderate resolution imaging spectroradiometer (MODIS) dataset. The annual MODIS layers were generated based on a maximum value composite procedure in the App Store of the ENVI 5.3 package. Then, the layers of different years were averaged to obtain a single layer representing the ecological factor. Twenty soil variables, including pH, electrical conductivity, fresh water content, air-dried water content, organic matter, and total N, P, C, S, Si, K, Ca, Na, Mg, Al, Fe, Mn, Zn, Cu, and Pb, were obtained from published research by our research group [82]. The three topographic variables included altitude, aspect, and slope. Altitude was derived from data recorded during field sampling. Aspect and slope were derived from SRTM elevation data under a digital terrain model with a resolution of 2.5 arc-mins, and the values for sampling sites were calculated in ArcGIS ver. 10.4.1 (http://www.esri.com/software/arcgis/arcgis-for-desktop). To reduce multicollinearity among variables, the variance inflation factors (VIFs) were calculated for the 19 bioclimatic variables, five ecological factors, 20 soil variables, and three topographic variables using the vif function of ‘usdm’ in R software [83]. Environmental variables with a VIF > 10 were removed. Five bioclimatic variables, four ecological factors, six soil variables, and three topographic variables were retained (Additional file 24).

### Isolation by distance (IBD) and isolation by environment (IBE)

To test for IBD and IBE, we generated geographic and environmental distance matrices. The 18 environmental variables (Additional file 24) were subjected to principal component analysis using the prcomp function in R software. We selected the resulting first five principal component axes, which explained 84.72% of the environmental variance. The five principal component axes were used to calculate environmental distances (Euclidean distance) using the dist function in R software. To obtain geographic distance matrices, we calculated the geographic distance in kilometers among populations.

To investigate the roles of environmental and geographic factors in shaping genetic differentiation, we calculated the associations between pairwise *F*_ST_ among populations and environmental distance and geographic distance with the Mantel test using the mantel function of the R package ‘vegan’ (https://github.com/vegandevs/vegan/), and the significance was determined for 999 permutations.

### Ecological niche modeling

After removing duplicate records, a total of 60 records of *P. chienii*, including 15 for GX, six for HN, 11 for JX, and 28 for ZJ, were collected from field sampling, published resources, the Chinese Virtual Herbarium (CVH, http://www.cvh.ac.cn/), and the Global Biodiversity Information Facility (GBIF, http://www.gbif.org), which covered most of the distribution range of this species.

Current climatic data (averaging over the period 1950–2000) comprising 19 bioclimatic variables at a spatial resolution of 2.5 arc-mins were obtained from the WorldClim database (http://www.worldclim.org). The VIFs were calculated for the 19 bioclimatic variables to reduce the multicollinearity among the variables. Bioclimatic variables with a VIF > 10 were removed. Five bioclimatic variables were retained, namely, mean temperature of the coldest quarter, precipitation of the wettest month, precipitation of the driest month, precipitation seasonality (CV), and precipitation of warmest the quarter. Maxent ver. 3.3.3 k [84] was used to predict the current potential distributions of the four groups (ZJ, JX, GX, and HN) of *P. chienii* with the following parameters: replicates, 10; replicated run type, subsample; maximum iterations, 500; and random test points, 25. The AUC values were used to predict the performance of the models, with AUC values closer to 1.0 indicating better model performance. ENMTools ver. 1.4.4 [85] was used to measure the niche differences between pairs of the four groups using the niche overlap tool. Schoener’s *D* and the standardized Hellinger distance (*I*) were calculated to measure niche overlap in group pairs. Identity tests of six comparisons (GX vs. JX, GX vs. ZJ, HN vs. GX, HN vs. JX, HN vs. ZJ, and JX vs. ZJ) were performed.

To examine the effects of past and future climatic shifts on the four groups of *P. chienii*, ecological niche modeling was used to predict potential distribution patterns during the future (2050, average for 2041–2060), the LIG (approximately 130–114 kya), and the LGM (approximately 21 kya). Climate layers for the LGM and future at a spatial resolution of 2.5 arc-mins and LIG at a spatial resolution of 30 arc-secs were obtained from the WorldClim database.

### Detection of candidate unigenes and annotation

To determine if there were unigenes putatively under selection in the 10 populations, we implemented an *F*_ST_ outlier approach in BayeScan ver. 2.1 [86]. It has been reported that BayeScan software has lower false-positive rates than other similar software programs. This method is based on a logistic regression that decomposes genetic variation into a population-specific *F*_ST_ coefficient (β) shared by all loci and a locus-specific *F*_ST_ coefficient (α) shared by all the populations [86]. All parameters set in BayeScan software were kept as the default. To reduce the occurrence of false positives, we calculated the *q*-values in BayeScan, and SNPs with a *q*-value lower than 0.001 were considered outlier SNPs. We also calculated the locus-specific *F*_ST_ coefficient (α), where a positive value indicates diversifying selection, while a negative value indicates purifying/balancing selection. In the analysis of the correlation between genomic variation and environmental variables, we considered only SNPs with positive α values and ignored SNPs with negative α values.

Based on niche overlap analysis, the ecological differentiation of GX vs. ZJ and HN vs. ZJ was valid. Therefore, we further used selective sweep analysis to detect adaptation. We calculated the *F*_ST_ values and π ratios (π_GX_/π_ZJ_, π_ZJ_/π_GX_, π_HN_/π_ZJ_, and π_ZJ_/π_HN_) for group pairs. The π ratios were subjected to log_2_ transformation. The regions with *F*_ST_ and log_2_(π ratio) values in the top 5% were considered candidate outliers subjected to strong selective sweeps. Then, all outliers were assigned to corresponding unigenes.

We performed functional annotation of the candidate unigenes containing outlier SNPs identified in BayeScan and selective sweep analysis. Hmmscan of HMMER ver. 3.1 [87] was used to perform Pfam protein database annotation. Based on the protein annotation information from the Pfam database, GO term annotation was determined using Blast2GO [88] and a custom script. KEGG and Swiss-Prot database annotations were implemented using DIAMOND ver. 0.8.36 [89] with an *E*-value of 1.0 × 10^− 5^. To identify significantly enriched biological functions and pathways, we performed GO and KEGG enrichment analyses. The GO enrichment analysis of candidate unigenes was performed using the ‘GOseq’ R package based on the Wallenius noncentral hypergeometric distribution [90]. KEGG pathway enrichment was determined using KOBAS (2.0) [91]. *q*-values (adjusted *p*-values) were used to test the statistical significance of GO and KEGG enrichment, with *q*-values < 0.05 considered significant.

### Association of genomic variation with environmental variables

We utilized RDA, a multivariate method, to detect the relative importance of environmental and geographic variables to genetic variation. RDA, as a canonical ordination method, allows for calculation of the variance in response variables explained by a number of explanatory variables (canonical axes). To avoid spatial autocorrelation, longitude and latitude coordinates of each individual were transformed using distance-based Moran’s eigenvector maps (dbMEM1–dbMEM6, representing geographic variables). We used environmental variables (Additional file 24) and geographic variables (dbMEM1–dbMEM6) as explanatory variables. Hellinger-transformed allele data were used as response variables. To avoid overfitting, we performed forward selection on the environmental variables and geographic variables separately using the ordiR2step function of the R package ‘vegan’ [92] to remove variables lacking explanatory power for partitioning. The final retained environmental and geographic variables are shown in Additional file 21 for the following analyses. The SNP matrixes, including all loci and outlier loci, were used as response variables in RDA. Environmental and geographic explanatory variables and the SNP matrixes of response variables were used in RDA with no conditional treatment. We also performed variation partitioning in RDA using the varpart function of the R package ‘vegan’. All RDA models and constrained axes were assessed for significance with 999 permutations using the ANOVA.cca function in R software.

A univariate method for LFMM was also used to test correlations of outlier SNPs with environmental variables using the ‘LEA’ package in R [93]. LFMM is a powerful tool for testing the correlation of single-locus–single-variable after taking population structure into account, thereby accurately detecting adaptive signatures, and has been proven to strike a good balance between low false-positive rates and high power [94]. The number of latent factors (*K*) was set to 4 based on the ADMIXTURE result. The analysis was run with a burn-in of 100,000, 50,000 iterations, and 10 repetitions. The *p*-values were used to test for statistical significance, with *p*-values < 0.005 indicating significant correlations. Candidate unigenes that were significantly related to environmental variables were subjected to functional annotation.

## Supplementary Information

**Additional file 1 **Summary of the reads sequenced for 108 *Pseudotaxus chienii* individuals.

**Additional file 2 **Summary of the unigenes for *Pseudotaxus chienii*.

**Additional file 3.** The length distribution of unigenes.

**Additional file 4.** The mapping rates between the clean reads of each individual and the reference sequences.

**Additional file 5 **The pairwise *F*_ST_ values between the 10 *Pseudotaxus chienii* populations.

**Additional file 6 **The pairwise *F*_ST_ values between the four *Pseudotaxus chienii* groups.

**Additional file 7 **Gene expression analysis based on FPKM data in *Pseudotaxus chienii*. (a) The distribution of population gene expression (*E*_p_). (b) The distribution of expression diversity (*E*_d_).

**Additional file 8 **The relationship between nucleotide diversity (π) and expression diversity (*E*_d_) of unigenes in each population.

**Additional file 9 **The relationship between nucleotide diversity (π) and population gene expression level (*E*_p_) among 10 populations.

**Additional file 10 **The relationship between population expression similarity (*E*_p_ similarity) and genetic distance.

**Additional file 11.** Independent contributions of five bioclimatic variables for four groups.

**Additional file 12 **Predicted potential distributions for the four groups of *Pseudotaxus chienii* (ZJ, JX, GX, and HN). (a) Present day. (b) Last interglacial (LIG, c. 120–140 kya). (c) Last glacial maximum (LGM, c. 21 kya). (d) Future (2050, average for 2041–2060).

**Additional file 13.** The annotation of the unigenes containing outlier SNPs.

**Additional file 14.** Gene ontology (GO) annotation of the candidate unigenes containing outlier SNPs identified in BayeScan.

**Additional file 15.** Gene ontology (GO) enrichment analysis of 642 unigenes containing outlier SNPs.

**Additional file 16.** The annotation of the 87 candidate unigenes for Zhejiang (ZJ) group habitat adaptation.

**Additional file 17.** Kyoto Encyclopedia of Genes and Genomes (KEGG) pathway enrichment analysis of the 87 candidate unigenes for habitat adaptation in the Zhejiang (ZJ) group.

**Additional file 18.** The annotation of the three candidate unigenes for habitat adaptation in the Hunan (HN) group.

**Additional file 19.** Kyoto Encyclopedia of Genes and Genomes (KEGG) pathway enrichment analysis of three candidate unigenes for habitat adaptation in the Hunan (HN) group.

**Additional file 20.** The annotation of the 17 candidate unigenes for habitat adaptation in the Guangxi (GX) group.

**Additional file 21.** The significant environmental and geographic variables retained by the initial step-forward selection method for all loci and outlier loci.

**Additional file 22.** Outlier SNPs associated with environmental variables identified using LFMM.

**Additional file 23.** The annotation of the unigenes containing outlier SNPs associated with environmental variables.

**Additional file 24 **The environmental variables with variance inflation factors (VIFs) < 10 for 10 *Pseudotaxus chienii* populations.

## Data Availability

The raw RNA-Seq data are available from the Sequence Read Archive of the National Center for Biotechnology Information under the following: SRR12970057–SRR12970164.

## Abbreviations

SNP: Single nucleotide polymorphisms
*F*_ST_: Pairwise genetic differentiation values
*F*_IS_: Wright’s inbreeding coefficient
AMOVA: Analysis of molecular variance
IBD: Isolation by distance
IBE: Isolation by environment
PCA: Principal component analysis
AUC: Area under the receiver operating characteristic curve
GO: Gene ontology
KEGG: Kyoto Encyclopedia of Genes and Genomes
RDA: Redundancy analysis
LFMMs: Latent factor mixed models
