# Supplementary material for: Population transcriptomic sequencing reveals allopatric divergence and local adaptation in Pseudotaxus chienii (Taxaceae)
Source: BMC Genomics. 2021 May 26;22:388. doi: 10.1186/s12864-021-07682-3 (PMC8157689; doi:10.1186/s12864-021-07682-3)
Supplement: Supplementary file 1 — Additional file 1 Summary of the reads sequenced for 108 Pseudotaxus chienii individuals. [file 12864_2021_7682_MOESM1_ESM.docx]

**Additional file 1.** Summary of the reads sequenced for 108 *Pseudotaxus chienii* individuals.

| **Sample** | **Raw reads** | **Clean reads** | **Clean bases (G)** | **Error (%)** | **Q20 (%)** | **Q30 (%)** | **GC (%)** |
| --- | --- | --- | --- | --- | --- | --- | --- |
| BJS_1 | 62,393,118 | 61,733,488 | 9.26 | 0.02 | 98.2 | 94.57 | 45.92 |
| BJS_10 | 63,795,354 | 63,232,060 | 9.48 | 0.02 | 98.15 | 94.45 | 46.15 |
| BJS_11 | 57,032,078 | 56,161,116 | 8.42 | 0.02 | 98.1 | 94.42 | 46.05 |
| BJS_12 | 60,190,556 | 59,163,432 | 8.87 | 0.02 | 98.26 | 94.71 | 46.09 |
| BJS_2 | 65,838,684 | 65,188,044 | 9.78 | 0.02 | 98.35 | 94.95 | 45.76 |
| BJS_3 | 58,738,990 | 58,283,450 | 8.74 | 0.02 | 98.23 | 94.66 | 46.2 |
| BJS_4 | 60,942,124 | 60,335,128 | 9.05 | 0.02 | 98.2 | 94.6 | 45.65 |
| BJS_5 | 62,441,016 | 61,761,518 | 9.26 | 0.02 | 98.15 | 94.49 | 46.38 |
| BJS_6 | 64,289,518 | 63,632,000 | 9.54 | 0.03 | 98.03 | 94.2 | 46.17 |
| BJS_7 | 55,796,506 | 55,141,378 | 8.27 | 0.02 | 98.19 | 94.56 | 46.03 |
| BJS_8 | 59,398,448 | 58,804,040 | 8.82 | 0.02 | 98.22 | 94.63 | 46.26 |
| BJS_9 | 54,312,456 | 53,784,486 | 8.07 | 0.02 | 98.2 | 94.58 | 46.34 |
| DXG_1 | 68,210,648 | 66,930,962 | 10.04 | 0.02 | 98.31 | 94.85 | 46.06 |
| DXG_10 | 67,765,514 | 67,065,436 | 10.06 | 0.02 | 98.16 | 94.47 | 46.15 |
| DXG_11 | 65,156,034 | 64,352,192 | 9.65 | 0.03 | 98.02 | 94.12 | 46.17 |
| DXG_12 | 66,175,172 | 65,442,642 | 9.82 | 0.02 | 98.07 | 94.23 | 45.97 |
| DXG_2 | 68,348,264 | 67,429,302 | 10.11 | 0.02 | 98.24 | 94.68 | 46.37 |
| DXG_3 | 58,353,230 | 57,685,792 | 8.65 | 0.02 | 98.26 | 94.74 | 46.52 |
| DXG_4 | 67,680,196 | 66,718,350 | 10.01 | 0.02 | 98.15 | 94.49 | 46.35 |
| DXG_5 | 76,157,500 | 75,361,928 | 11.3 | 0.03 | 98.03 | 94.17 | 46.16 |
| DXG_6 | 70,449,382 | 69,713,344 | 10.46 | 0.02 | 98.24 | 94.67 | 46.01 |
| DXG_7 | 59,602,900 | 58,554,368 | 8.78 | 0.03 | 97.56 | 93 | 44.93 |
| DXG_8 | 66,170,474 | 65,556,662 | 9.83 | 0.03 | 97.35 | 92.55 | 45.92 |
| DXG_9 | 66,077,120 | 65,427,496 | 9.81 | 0.02 | 98.25 | 94.71 | 46.01 |
| LHS_1 | 59,687,700 | 58,991,020 | 8.85 | 0.02 | 98.21 | 94.57 | 45.57 |
| LHS_10 | 66,976,560 | 66,445,434 | 9.97 | 0.02 | 98.08 | 94.28 | 45.91 |
| LHS_11 | 59,582,116 | 59,131,178 | 8.87 | 0.02 | 98.18 | 94.49 | 46.16 |
| LHS_12 | 57,673,964 | 57,224,864 | 8.58 | 0.02 | 98.07 | 94.24 | 46.09 |
| LHS_2 | 56,502,542 | 55,880,122 | 8.38 | 0.02 | 98.21 | 94.6 | 45.77 |
| LHS_3 | 66,202,344 | 65,562,708 | 9.83 | 0.02 | 98.13 | 94.35 | 45.65 |
| LHS_4 | 80,680,394 | 79,922,164 | 11.99 | 0.02 | 98.26 | 94.67 | 45.75 |
| LHS_5 | 67,017,442 | 66,244,230 | 9.94 | 0.02 | 98.17 | 94.5 | 46.2 |
| LHS_6 | 71,344,364 | 70,581,968 | 10.59 | 0.02 | 98.17 | 94.5 | 45.88 |
| LHS_7 | 66,641,430 | 65,999,578 | 9.9 | 0.02 | 98.06 | 94.27 | 46.32 |
| LHS_8 | 62,145,968 | 61,550,204 | 9.23 | 0.02 | 98.07 | 94.26 | 46.23 |
| LHS_9 | 61,491,732 | 60,978,446 | 9.15 | 0.03 | 97.99 | 94.07 | 45.88 |
| LMD_1 | 51,257,434 | 50,495,286 | 7.57 | 0.03 | 97.45 | 92.78 | 44.74 |
| LMD_10 | 67,016,964 | 66,210,510 | 9.93 | 0.02 | 98.2 | 94.61 | 46.73 |
| LMD_11 | 69,681,392 | 69,030,596 | 10.35 | 0.03 | 98.05 | 94.19 | 45.98 |
| LMD_12 | 59,371,482 | 58,805,122 | 8.82 | 0.02 | 98.1 | 94.3 | 45.41 |
| LMD_2 | 54,046,714 | 53,100,364 | 7.97 | 0.03 | 97.59 | 93.08 | 44.64 |
| LMD_3 | 42,091,902 | 41,235,280 | 6.19 | 0.03 | 97.81 | 93.59 | 45.44 |
| LMD_4 | 58,478,204 | 58,010,772 | 8.7 | 0.02 | 98.22 | 94.63 | 46.53 |
| LMD_5 | 46,680,618 | 45,727,748 | 6.86 | 0.03 | 97.61 | 93.14 | 45.22 |
| LMD_6 | 68,054,396 | 67,442,798 | 10.12 | 0.02 | 98.26 | 94.7 | 44.89 |
| LMD_7 | 61,697,910 | 60,570,498 | 9.09 | 0.02 | 98.18 | 94.55 | 45.71 |
| LMD_8 | 63,087,202 | 62,184,018 | 9.33 | 0.02 | 98.29 | 94.79 | 45.64 |
| MS_1 | 65,620,022 | 64,990,264 | 9.75 | 0.02 | 98.27 | 94.73 | 46.12 |
| MS_10 | 54,640,958 | 53,910,730 | 8.09 | 0.02 | 98.23 | 94.63 | 45.66 |
| MS_11 | 60,602,842 | 60,029,476 | 9 | 0.02 | 98.19 | 94.55 | 46.13 |
| MS_12 | 59,099,020 | 58,208,610 | 8.73 | 0.02 | 98.33 | 94.89 | 45.92 |
| MS_2 | 60,862,040 | 60,433,920 | 9.07 | 0.03 | 97.41 | 92.43 | 46.17 |
| MS_3 | 56,646,434 | 56,217,222 | 8.43 | 0.02 | 98.08 | 94.27 | 46.14 |
| MS_4 | 58,287,992 | 57,758,588 | 8.66 | 0.02 | 98.14 | 94.43 | 46.17 |
| MS_5 | 64,215,922 | 63,718,332 | 9.56 | 0.02 | 98.18 | 94.49 | 45.92 |
| MS_6 | 54,964,730 | 54,485,346 | 8.17 | 0.02 | 98.06 | 94.21 | 45.95 |
| MS_7 | 64,696,754 | 64,246,408 | 9.64 | 0.02 | 98.28 | 94.78 | 46.07 |
| MS_8 | 57,387,966 | 56,931,692 | 8.54 | 0.02 | 98.29 | 94.73 | 45.78 |
| MS_9 | 62,431,974 | 61,473,760 | 9.22 | 0.02 | 98.2 | 94.53 | 46.2 |
| SMJ_1 | 64,439,006 | 63,403,886 | 9.51 | 0.02 | 98.1 | 94.35 | 45.45 |
| SMJ_10 | 50,509,800 | 49,709,166 | 7.46 | 0.02 | 98.2 | 94.58 | 46.73 |
| SMJ_11 | 55,342,136 | 54,511,402 | 8.18 | 0.02 | 98.19 | 94.56 | 46.19 |
| SMJ_12 | 51,120,316 | 49,759,800 | 7.46 | 0.02 | 98.22 | 94.73 | 46.93 |
| SMJ_2 | 64,757,244 | 63,986,300 | 9.6 | 0.02 | 98.05 | 94.23 | 46 |
| SMJ_3 | 49,148,048 | 48,585,314 | 7.29 | 0.02 | 98.05 | 94.21 | 46.54 |
| SMJ_4 | 53,952,542 | 52,743,284 | 7.91 | 0.03 | 97.93 | 94.06 | 46.55 |
| SMJ_5 | 56,082,330 | 55,289,416 | 8.29 | 0.03 | 97.98 | 94.12 | 46.51 |
| SMJ_6 | 48,966,386 | 48,070,180 | 7.21 | 0.03 | 97.76 | 93.46 | 44.59 |
| SMJ_7 | 58,098,616 | 57,375,724 | 8.61 | 0.02 | 98.23 | 94.72 | 46.35 |
| SMJ_8 | 51,942,738 | 51,213,178 | 7.68 | 0.03 | 98.02 | 94.21 | 46.54 |
| SMJ_9 | 58,918,662 | 57,962,910 | 8.69 | 0.02 | 98.16 | 94.5 | 46.35 |
| SQS_1 | 54,209,480 | 53,495,990 | 8.02 | 0.02 | 98.19 | 94.57 | 46.12 |
| SQS_2 | 60,493,364 | 59,872,986 | 8.98 | 0.03 | 97.97 | 94.01 | 45.97 |
| SQS_3 | 57,715,226 | 57,043,328 | 8.56 | 0.03 | 97.99 | 94.1 | 46.52 |
| SQS_4 | 57,956,652 | 57,357,344 | 8.6 | 0.02 | 98.19 | 94.55 | 45.61 |
| SQS_5 | 51,364,786 | 50,868,628 | 7.63 | 0.03 | 97.32 | 92.22 | 46.14 |
| SQS_6 | 54,357,622 | 53,803,196 | 8.07 | 0.03 | 97.99 | 94.12 | 46.11 |
| SQS_7 | 57,599,242 | 56,938,746 | 8.54 | 0.02 | 98.04 | 94.27 | 46.12 |
| SQS_8 | 51,704,232 | 51,175,936 | 7.68 | 0.02 | 98.09 | 94.34 | 46.42 |
| YSGY_1 | 54,633,298 | 54,012,540 | 8.1 | 0.03 | 97.91 | 93.88 | 45.92 |
| YSGY_10 | 59,202,486 | 58,521,770 | 8.78 | 0.02 | 98.16 | 94.5 | 45.97 |
| YSGY_11 | 57,265,962 | 56,534,000 | 8.48 | 0.02 | 98.1 | 94.38 | 46 |
| YSGY_12 | 57,764,898 | 57,148,902 | 8.57 | 0.02 | 98.11 | 94.38 | 45.79 |
| YSGY_2 | 47,946,490 | 47,266,996 | 7.09 | 0.02 | 98.08 | 94.34 | 46.14 |
| YSGY_3 | 46,471,056 | 45,797,930 | 6.87 | 0.02 | 98.21 | 94.6 | 45.85 |
| YSGY_4 | 49,521,270 | 48,704,754 | 7.31 | 0.02 | 98.06 | 94.31 | 46.18 |
| YSGY_5 | 51,828,258 | 51,272,466 | 7.69 | 0.02 | 98.19 | 94.58 | 45.73 |
| YSGY_6 | 61,337,680 | 60,583,536 | 9.09 | 0.02 | 98.08 | 94.31 | 45.66 |
| YSGY_7 | 62,137,136 | 61,486,404 | 9.22 | 0.02 | 98.24 | 94.71 | 45.99 |
| YSGY_8 | 51,203,548 | 50,590,454 | 7.59 | 0.02 | 98.25 | 94.72 | 46.28 |
| YSGY_9 | 58,342,538 | 57,010,652 | 8.55 | 0.02 | 98.07 | 94.32 | 45.54 |
| ZJJ_1 | 54,479,112 | 53,712,490 | 8.06 | 0.02 | 98.05 | 94.27 | 46.66 |
| ZJJ_2 | 47,539,750 | 46,758,812 | 7.01 | 0.02 | 98.26 | 94.74 | 46.06 |
| ZJJ_3 | 54,579,228 | 53,657,442 | 8.05 | 0.02 | 98.21 | 94.59 | 45.67 |
| ZJJ_4 | 46,290,752 | 45,499,316 | 6.82 | 0.03 | 97.96 | 94.03 | 46.09 |
| ZJJ_5 | 54,471,242 | 53,783,666 | 8.07 | 0.02 | 98.23 | 94.66 | 45.38 |
| ZZB_1 | 51,442,104 | 50,766,754 | 7.62 | 0.03 | 98.02 | 94.18 | 46.31 |
| ZZB_10 | 54,144,034 | 53,387,776 | 8.01 | 0.02 | 98.18 | 94.55 | 46 |
| ZZB_11 | 59,150,276 | 58,270,674 | 8.74 | 0.03 | 97.98 | 94.07 | 45.95 |
| ZZB_12 | 51,699,626 | 51,059,378 | 7.66 | 0.02 | 98.03 | 94.24 | 46.49 |
| ZZB_2 | 43,702,604 | 43,118,308 | 6.47 | 0.02 | 98.17 | 94.5 | 46.12 |
| ZZB_3 | 55,759,932 | 54,954,428 | 8.24 | 0.02 | 98.21 | 94.6 | 46.29 |
| ZZB_4 | 51,996,578 | 51,186,840 | 7.68 | 0.02 | 98.18 | 94.59 | 45.97 |
| ZZB_5 | 54,642,522 | 53,885,398 | 8.08 | 0.03 | 97.99 | 94.11 | 46.19 |
| ZZB_6 | 62,854,138 | 61,984,092 | 9.3 | 0.02 | 98.12 | 94.39 | 45.77 |
| ZZB_7 | 53,275,758 | 52,476,996 | 7.87 | 0.03 | 98.04 | 94.19 | 45.92 |
| ZZB_8 | 51,520,980 | 50,882,794 | 7.63 | 0.02 | 98.08 | 94.32 | 46.22 |
| ZZB_9 | 60,387,034 | 59,471,174 | 8.92 | 0.02 | 98.05 | 94.23 | 46.13 |
| Total/Average | 6,336,451,428 | 6,258,141,326 | 938.69 | 0.02 | 98.09 | 94.32 | 46.00 |

Samples refer to individuals of populations in Table 1. Q20 percentage is proportion of nucleotides with quality value larger than 20. Q30 percentage is proportion of nucleotides with quality value larger than 30. GC percentage is proportion of guanidine (G) and cytosine (C) nucleotides among total nucleotides.
