# Supplementary material for: Population transcriptomic sequencing reveals allopatric divergence and local adaptation in Pseudotaxus chienii (Taxaceae)
Source: BMC Genomics. 2021 May 26;22:388. doi: 10.1186/s12864-021-07682-3 (PMC8157689; doi:10.1186/s12864-021-07682-3)
Supplement: Supplementary file 2 — Additional file 2 Summary of the unigenes for Pseudotaxus chienii. [file 12864_2021_7682_MOESM2_ESM.docx]

**Additional file 2.** Summary of the unigenes for *Pseudotaxus chienii*.

|  | **Min length (bp)** | **Mean length (bp)** | **Max length (bp)** | **N50 (bp)** | **Total nucleotide bases (bp)** | **Total number** |
| --- | --- | --- | --- | --- | --- | --- |
| Unigenes | 201 | 711 | 62,978 | 891 | 426,745,889 | 600,273 |

N50 means that the unigene size such that 50% of the entire assembly is contained in unigenes equal to or longer than this value.
