# Supplementary figures and images for: Population transcriptomic sequencing reveals allopatric divergence and local adaptation in Pseudotaxus chienii (Taxaceae)
Source: BMC Genomics. 2021 May 26;22:388. doi: 10.1186/s12864-021-07682-3 (PMC8157689; doi:10.1186/s12864-021-07682-3)

**Additional file 3.** The length distribution of unigenes.

**
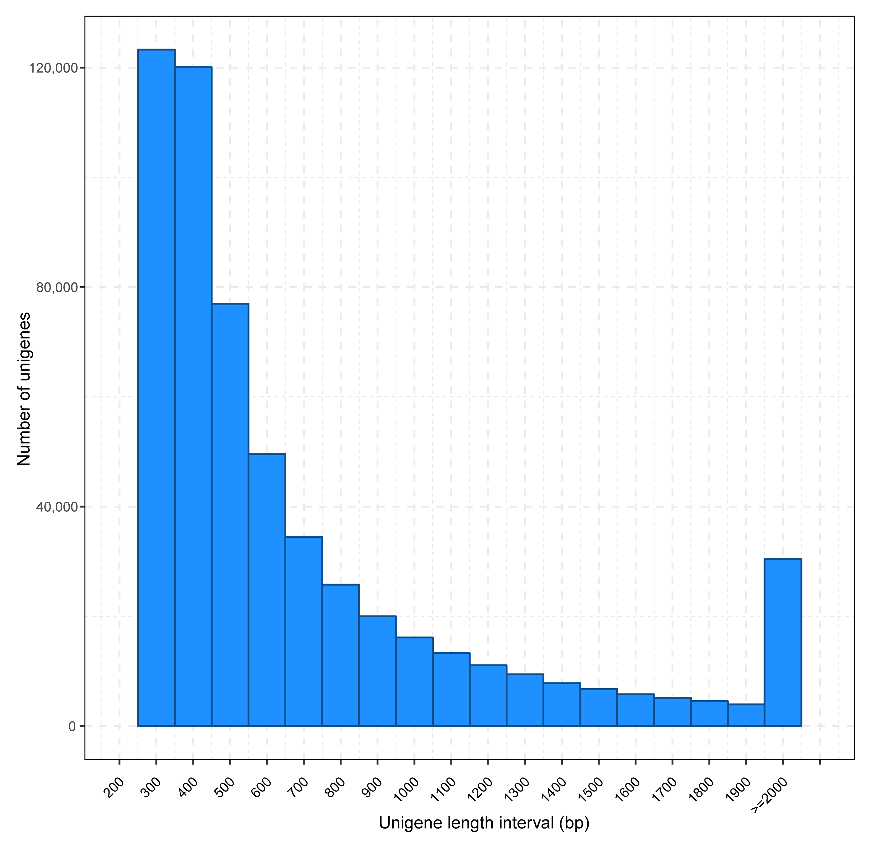
**

Supplement: Supplementary file 3 — Additional file 3. The length distribution of unigenes. [file 12864_2021_7682_MOESM3_ESM.docx]

**Additional file 15.** Gene ontology (GO) enrichment analysis of 642 unigenes containing outlier SNPs.


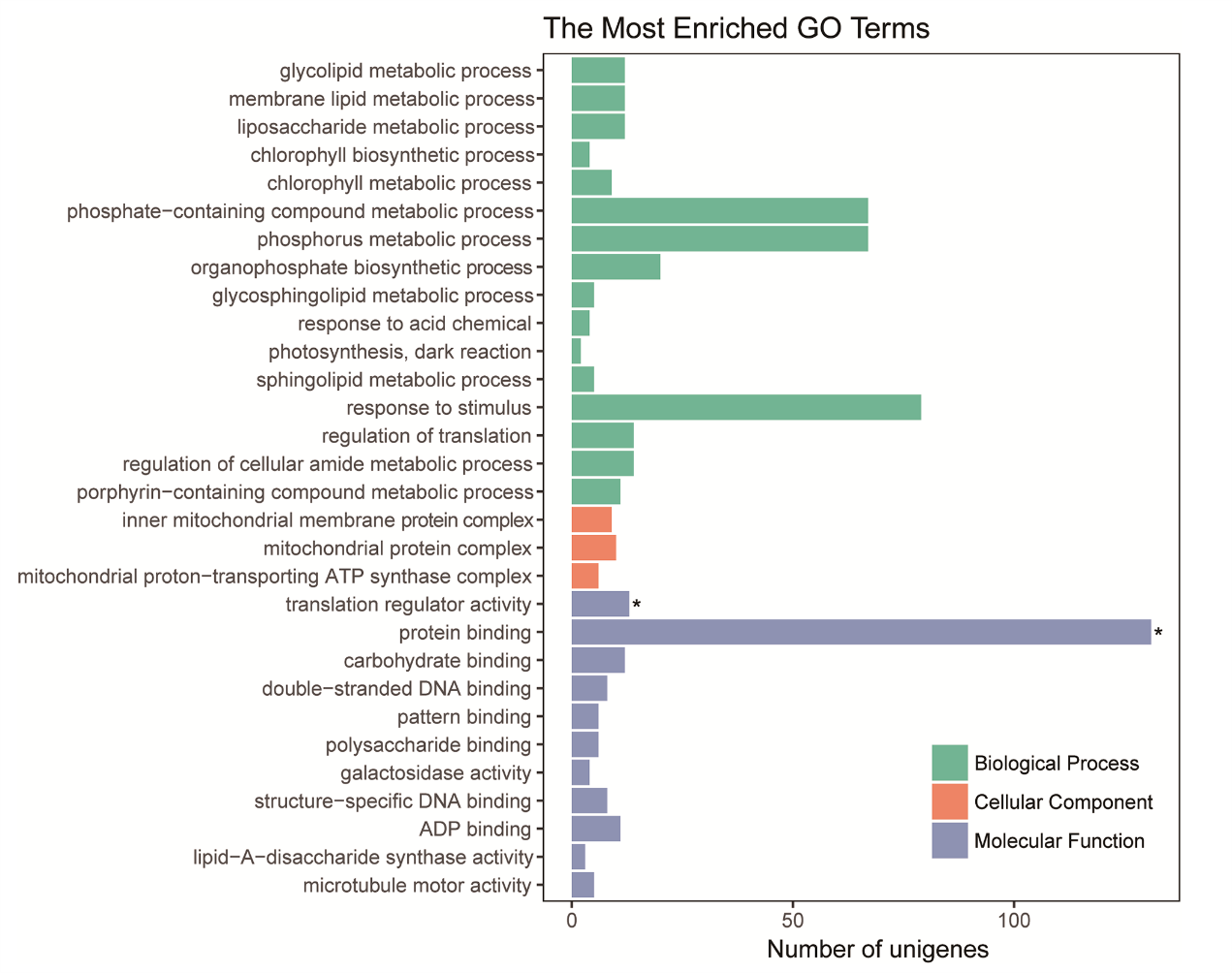

Supplement: Supplementary file 15 — Additional file 15. Gene ontology (GO) enrichment analysis of 642 unigenes containing outlier SNPs. [file 12864_2021_7682_MOESM15_ESM.docx]
