# Supplementary material for: Population transcriptomic sequencing reveals allopatric divergence and local adaptation in Pseudotaxus chienii (Taxaceae)
Source: BMC Genomics. 2021 May 26;22:388. doi: 10.1186/s12864-021-07682-3 (PMC8157689; doi:10.1186/s12864-021-07682-3)
Supplement: Supplementary file 4 — Additional file 4. The mapping rates between the clean reads of each individual and the reference sequences. [file 12864_2021_7682_MOESM4_ESM.docx]

**Additional file 4.** The mapping rates between the clean reads of each individual and the reference sequences.

| **Sample** | **Total reads** | **Total mapped** | **Mapping rates** |
| --- | --- | --- | --- |
| BJS_1 | 61,733,488 | 43,557,020 | 70.56% |
| BJS_10 | 63,232,060 | 44,048,826 | 69.66% |
| BJS_11 | 56,161,116 | 39,631,362 | 70.57% |
| BJS_12 | 59,163,432 | 41,290,280 | 69.79% |
| BJS_2 | 65,188,044 | 46,259,690 | 70.96% |
| BJS_3 | 58,283,450 | 40,702,792 | 69.84% |
| BJS_4 | 60,335,128 | 42,543,652 | 70.51% |
| BJS_5 | 61,761,518 | 43,114,024 | 69.81% |
| BJS_6 | 63,632,000 | 44,289,592 | 69.60% |
| BJS_7 | 55,141,378 | 38,884,252 | 70.52% |
| BJS_8 | 58,804,040 | 39,737,134 | 67.58% |
| BJS_9 | 53,784,486 | 37,707,528 | 70.11% |
| DXG_1 | 66,930,962 | 48,704,376 | 72.77% |
| DXG_10 | 67,065,436 | 48,028,304 | 71.61% |
| DXG_11 | 64,352,192 | 45,243,042 | 70.31% |
| DXG_12 | 65,442,642 | 46,934,952 | 71.72% |
| DXG_2 | 67,429,302 | 49,027,942 | 72.71% |
| DXG_3 | 57,685,792 | 41,587,724 | 72.09% |
| DXG_4 | 66,718,350 | 46,838,664 | 70.20% |
| DXG_5 | 75,361,928 | 53,602,378 | 71.13% |
| DXG_6 | 69,713,344 | 49,772,166 | 71.40% |
| DXG_7 | 58,554,368 | 43,420,722 | 74.15% |
| DXG_8 | 65,556,662 | 46,185,602 | 70.45% |
| DXG_9 | 65,427,496 | 46,196,128 | 70.61% |
| LHS_1 | 58,991,020 | 41,894,276 | 71.02% |
| LHS_10 | 66,445,434 | 46,634,622 | 70.18% |
| LHS_11 | 59,131,178 | 41,019,310 | 69.37% |
| LHS_12 | 57,224,864 | 40,009,772 | 69.92% |
| LHS_2 | 55,880,122 | 40,040,458 | 71.65% |
| LHS_3 | 65,562,708 | 46,631,046 | 71.12% |
| LHS_4 | 79,922,164 | 57,338,386 | 71.74% |
| LHS_5 | 66,244,230 | 46,429,424 | 70.09% |
| LHS_6 | 70,581,968 | 49,984,616 | 70.82% |
| LHS_7 | 65,999,578 | 45,774,622 | 69.36% |
| LHS_8 | 61,550,204 | 42,562,258 | 69.15% |
| LHS_9 | 60,978,446 | 42,606,596 | 69.87% |
| LMD_1 | 50,495,286 | 36,813,490 | 72.90% |
| LMD_10 | 66,210,510 | 44,017,168 | 66.48% |
| LMD_11 | 69,030,596 | 47,747,604 | 69.17% |
| LMD_12 | 58,805,122 | 40,418,598 | 68.73% |
| LMD_2 | 53,100,364 | 38,379,126 | 72.28% |
| LMD_3 | 41,235,280 | 29,780,976 | 72.22% |
| LMD_4 | 58,010,772 | 38,729,332 | 66.76% |
| LMD_5 | 45,727,748 | 33,631,104 | 73.55% |
| LMD_6 | 67,442,798 | 46,915,258 | 69.56% |
| LMD_7 | 60,570,498 | 43,125,280 | 71.20% |
| LMD_8 | 62,184,018 | 43,351,608 | 69.72% |
| MS_1 | 64,990,264 | 44,828,378 | 68.98% |
| MS_10 | 53,910,730 | 38,631,696 | 71.66% |
| MS_11 | 60,029,476 | 42,397,410 | 70.63% |
| MS_12 | 58,208,610 | 42,543,136 | 73.09% |
| MS_2 | 60,433,920 | 42,131,706 | 69.72% |
| MS_3 | 56,217,222 | 38,516,240 | 68.51% |
| MS_4 | 57,758,588 | 40,659,008 | 70.39% |
| MS_5 | 63,718,332 | 44,905,538 | 70.48% |
| MS_6 | 54,485,346 | 38,706,256 | 71.04% |
| MS_7 | 64,246,408 | 45,587,164 | 70.96% |
| MS_8 | 56,931,692 | 40,129,624 | 70.49% |
| MS_9 | 61,473,760 | 44,275,664 | 72.02% |
| SMJ_1 | 63,403,886 | 44,637,742 | 70.40% |
| SMJ_10 | 49,709,166 | 35,645,950 | 71.71% |
| SMJ_11 | 54,511,402 | 38,763,846 | 71.11% |
| SMJ_12 | 49,759,800 | 35,157,584 | 70.65% |
| SMJ_2 | 63,986,300 | 45,206,470 | 70.65% |
| SMJ_3 | 48,585,314 | 33,649,514 | 69.26% |
| SMJ_4 | 52,743,284 | 36,377,468 | 68.97% |
| SMJ_5 | 55,289,416 | 37,872,704 | 68.50% |
| SMJ_6 | 48,070,180 | 35,439,650 | 73.72% |
| SMJ_7 | 57,375,724 | 40,520,248 | 70.62% |
| SMJ_8 | 51,213,178 | 36,380,744 | 71.04% |
| SMJ_9 | 57,962,910 | 40,325,076 | 69.57% |
| SQS_1 | 53,495,990 | 37,538,954 | 70.17% |
| SQS_2 | 59,872,986 | 41,103,778 | 68.65% |
| SQS_3 | 57,043,328 | 39,628,288 | 69.47% |
| SQS_4 | 57,357,344 | 40,424,822 | 70.48% |
| SQS_5 | 50,868,628 | 35,201,784 | 69.20% |
| SQS_6 | 53,803,196 | 37,536,170 | 69.77% |
| SQS_7 | 56,938,746 | 38,747,800 | 68.05% |
| SQS_8 | 51,175,936 | 35,650,630 | 69.66% |
| YSGY_1 | 54,012,540 | 37,495,282 | 69.42% |
| YSGY_10 | 58,521,770 | 40,936,966 | 69.95% |
| YSGY_11 | 56,534,000 | 39,663,222 | 70.16% |
| YSGY_12 | 57,148,902 | 40,428,650 | 70.74% |
| YSGY_2 | 47,266,996 | 33,001,994 | 69.82% |
| YSGY_3 | 45,797,930 | 31,996,314 | 69.86% |
| YSGY_4 | 48,704,754 | 33,794,922 | 69.39% |
| YSGY_5 | 51,272,466 | 36,408,172 | 71.01% |
| YSGY_6 | 60,583,536 | 42,709,102 | 70.50% |
| YSGY_7 | 61,486,404 | 43,839,492 | 71.30% |
| YSGY_8 | 50,590,454 | 35,443,346 | 70.06% |
| YSGY_9 | 57,010,652 | 40,179,584 | 70.48% |
| ZJJ_1 | 53,712,490 | 37,758,406 | 70.30% |
| ZJJ_2 | 46,758,812 | 32,649,586 | 69.83% |
| ZJJ_3 | 53,657,442 | 37,715,436 | 70.29% |
| ZJJ_4 | 45,499,316 | 32,163,348 | 70.69% |
| ZJJ_5 | 53,783,666 | 37,948,430 | 70.56% |
| ZZB_1 | 50,766,754 | 35,986,828 | 70.89% |
| ZZB_10 | 53,387,776 | 37,738,128 | 70.69% |
| ZZB_11 | 58,270,674 | 41,641,324 | 71.46% |
| ZZB_12 | 51,059,378 | 35,534,284 | 69.59% |
| ZZB_2 | 43,118,308 | 30,607,104 | 70.98% |
| ZZB_3 | 54,954,428 | 39,296,000 | 71.51% |
| ZZB_4 | 51,186,840 | 36,712,340 | 71.72% |
| ZZB_5 | 53,885,398 | 37,935,800 | 70.40% |
| ZZB_6 | 61,984,092 | 43,747,754 | 70.58% |
| ZZB_7 | 52,476,996 | 37,346,950 | 71.17% |
| ZZB_8 | 50,882,794 | 35,887,008 | 70.53% |
| ZZB_9 | 59,471,174 | 41,475,066 | 69.74% |

Samples refer to individuals of populations in Table 1.
