# Supplementary material for: Population transcriptomic sequencing reveals allopatric divergence and local adaptation in Pseudotaxus chienii (Taxaceae)
Source: BMC Genomics. 2021 May 26;22:388. doi: 10.1186/s12864-021-07682-3 (PMC8157689; doi:10.1186/s12864-021-07682-3)
Supplement: Supplementary file 5 — Additional file 5 The pairwise FST values between the 10 Pseudotaxus chienii populations. [file 12864_2021_7682_MOESM5_ESM.docx]

**Additional file 5.** The pairwise F_ST_ values between the 10 *Pseudotaxus chienii* populations.

|  | **ZZB** | **BJS** | **SMJ** | **MS** | **DXG** | **LMD** | **SQS** | **YSGY** | **LHS** | **ZJJ** |
| --- | --- | --- | --- | --- | --- | --- | --- | --- | --- | --- |
| ZZB | 0 |  |  |  |  |  |  |  |  |  |
| BJS | -0.001 | 0 |  |  |  |  |  |  |  |  |
| SMJ | 0.242 | 0.251 | 0 |  |  |  |  |  |  |  |
| MS | 0.245 | 0.255 | 0.080 | 0 |  |  |  |  |  |  |
| DXG | 0.299 | 0.306 | 0.153 | 0.133 | 0 |  |  |  |  |  |
| LMD | 0.238 | 0.248 | -0.004 | 0.078 | 0.152 | 0 |  |  |  |  |
| SQS | 0.252 | 0.264 | 0.100 | 0.113 | 0.173 | 0.099 | 0 |  |  |  |
| YSGY | 0.328 | 0.337 | 0.280 | 0.287 | 0.347 | 0.280 | 0.295 | 0 |  |  |
| LHS | 0.353 | 0.361 | 0.309 | 0.308 | 0.365 | 0.309 | 0.317 | 0.176 | 0 |  |
| ZJJ | 0.367 | 0.380 | 0.300 | 0.300 | 0.363 | 0.318 | 0.310 | 0.349 | 0.373 | 0 |
