# Supplementary material for: Population transcriptomic sequencing reveals allopatric divergence and local adaptation in Pseudotaxus chienii (Taxaceae)
Source: BMC Genomics. 2021 May 26;22:388. doi: 10.1186/s12864-021-07682-3 (PMC8157689; doi:10.1186/s12864-021-07682-3)
Supplement: Supplementary file 6 — Additional file 6 The pairwise FST values between the four Pseudotaxus chienii groups. [file 12864_2021_7682_MOESM6_ESM.docx]

**Additional file 6.** The pairwise F_ST_ values between the four *Pseudotaxus chienii* groups.

|  | **JX** | **ZJ** | **GX** | **HN** |
| --- | --- | --- | --- | --- |
| JX | 0 |  |  |  |
| ZJ | 0.216 | 0 |  |  |
| GX | 0.311 | 0.231 | 0 |  |
| HN | 0.361 | 0.248 | 0.305 | 0 |

JX, Jiangxi group; ZJ, Zhejiang group; GX, Guangxi group; HN, Hunan group.
