# Supplementary material for: Population transcriptomic sequencing reveals allopatric divergence and local adaptation in Pseudotaxus chienii (Taxaceae)
Source: BMC Genomics. 2021 May 26;22:388. doi: 10.1186/s12864-021-07682-3 (PMC8157689; doi:10.1186/s12864-021-07682-3)

**Additional file 7.** Gene expression analysis based on FPKM data in *Pseudotaxus chienii*. (a) The distribution of population gene expression (*E*_p_). (b) The distribution of expression diversity (*E*_d_).


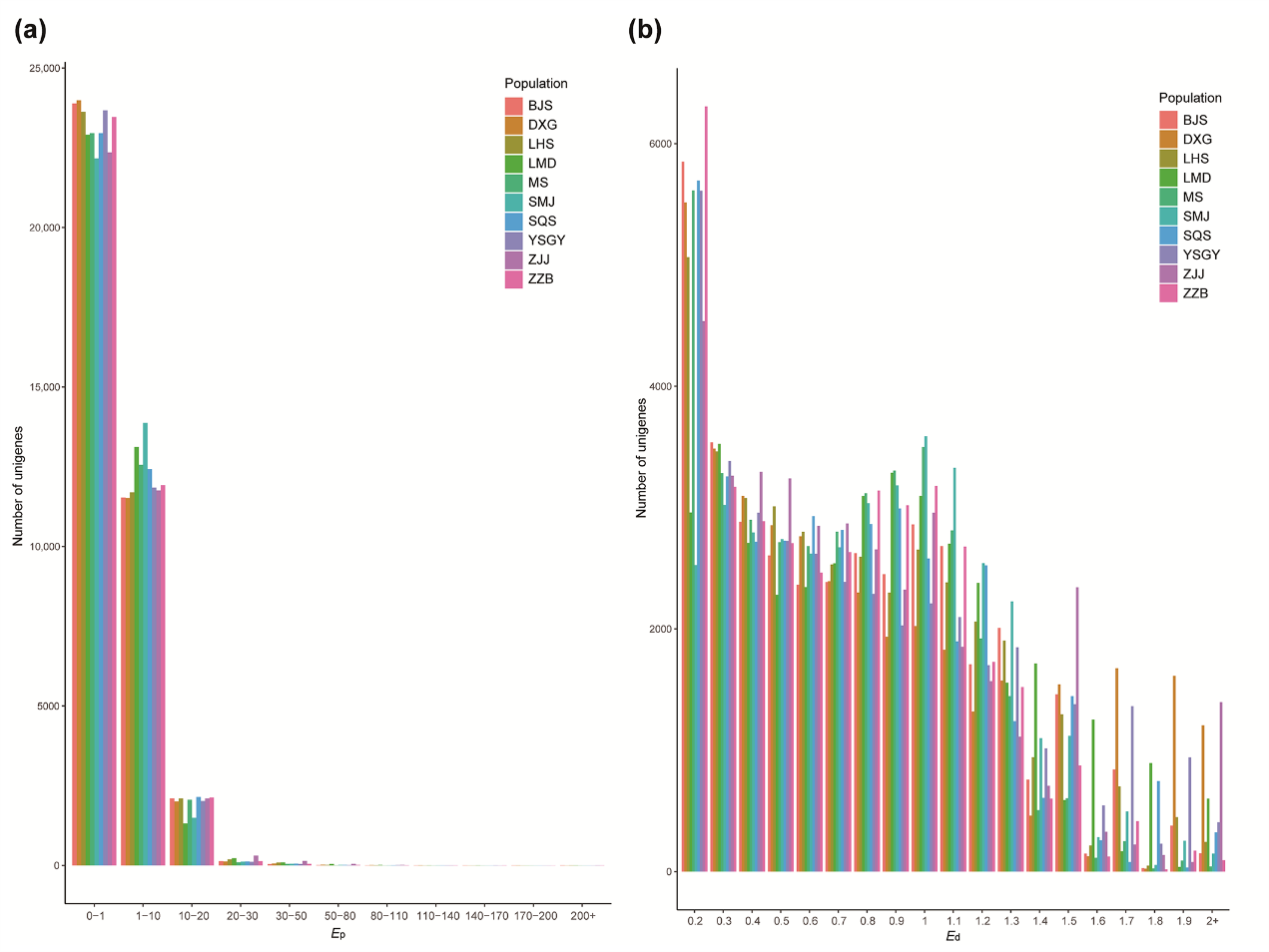

Supplement: Supplementary file 7 — Additional file 7 Gene expression analysis based on FPKM data in Pseudotaxus chienii. (a) The distribution of population gene expression (Ep). (b) The distribution of expression diversity (Ed). [file 12864_2021_7682_MOESM7_ESM.docx]
