# Supplementary material for: Population transcriptomic sequencing reveals allopatric divergence and local adaptation in Pseudotaxus chienii (Taxaceae)
Source: BMC Genomics. 2021 May 26;22:388. doi: 10.1186/s12864-021-07682-3 (PMC8157689; doi:10.1186/s12864-021-07682-3)

**Additional file 8.** The relationship between nucleotide diversity (π) and expression diversity (*E*_d_) of unigenes in each population.


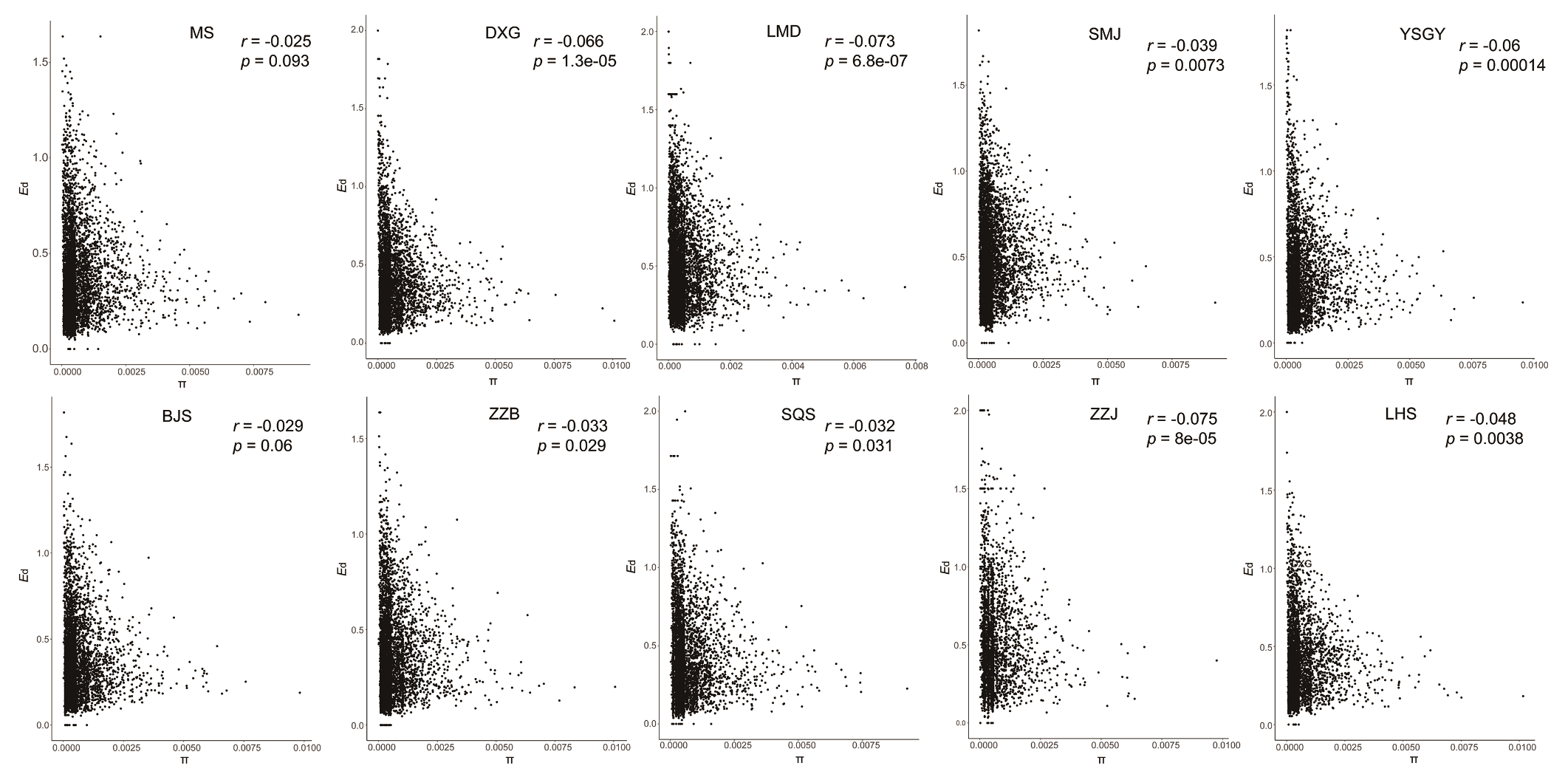

Supplement: Supplementary file 8 — Additional file 8 The relationship between nucleotide diversity (π) and expression diversity (Ed) of unigenes in each population. [file 12864_2021_7682_MOESM8_ESM.docx]
