# Supplementary material for: Population transcriptomic sequencing reveals allopatric divergence and local adaptation in Pseudotaxus chienii (Taxaceae)
Source: BMC Genomics. 2021 May 26;22:388. doi: 10.1186/s12864-021-07682-3 (PMC8157689; doi:10.1186/s12864-021-07682-3)

**Additional file 9.** The relationship between nucleotide diversity (π) and population gene expression level (*E*_p_) among 10 populations.


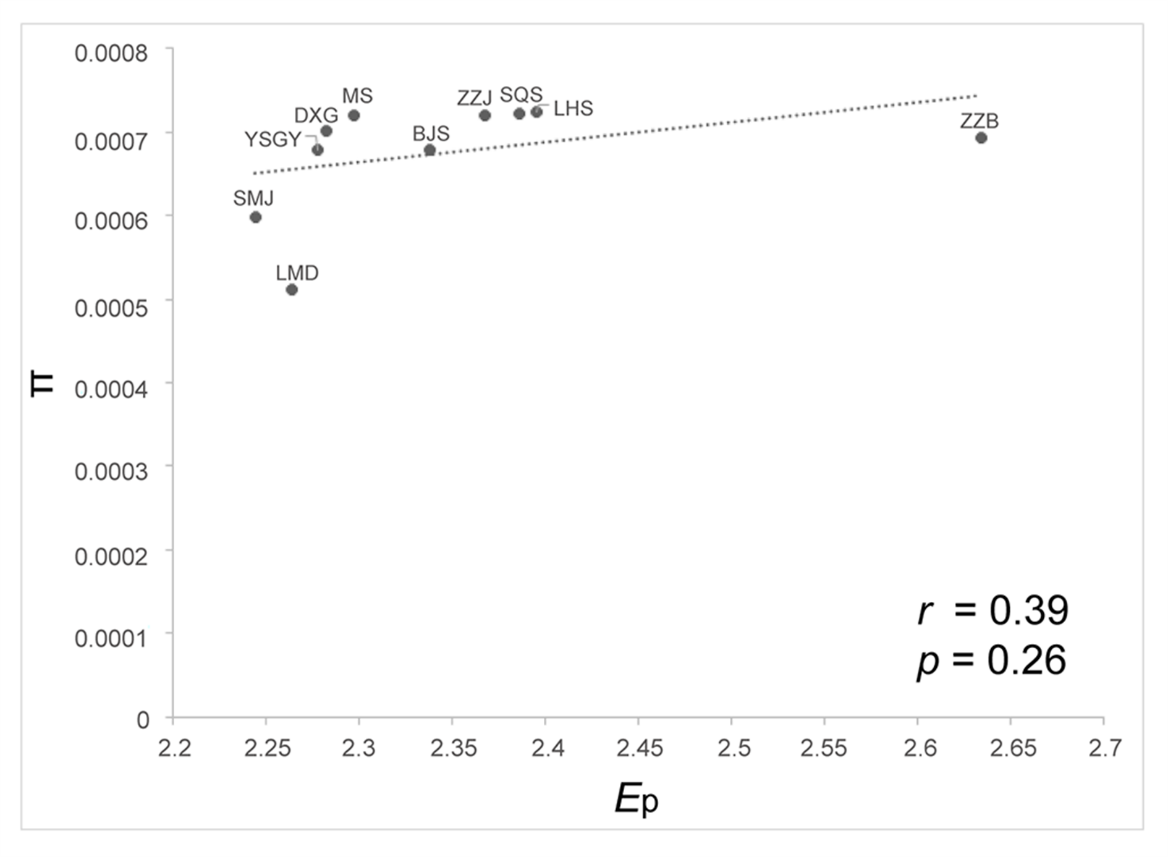

Supplement: Supplementary file 9 — Additional file 9 The relationship between nucleotide diversity (π) and population gene expression level (Ep) among 10 populations. [file 12864_2021_7682_MOESM9_ESM.docx]
