# Supplementary material for: Population transcriptomic sequencing reveals allopatric divergence and local adaptation in Pseudotaxus chienii (Taxaceae)
Source: BMC Genomics. 2021 May 26;22:388. doi: 10.1186/s12864-021-07682-3 (PMC8157689; doi:10.1186/s12864-021-07682-3)

**Additional file 10.** The relationship between population expression similarity (*E*_p_ similarity) and genetic distance.


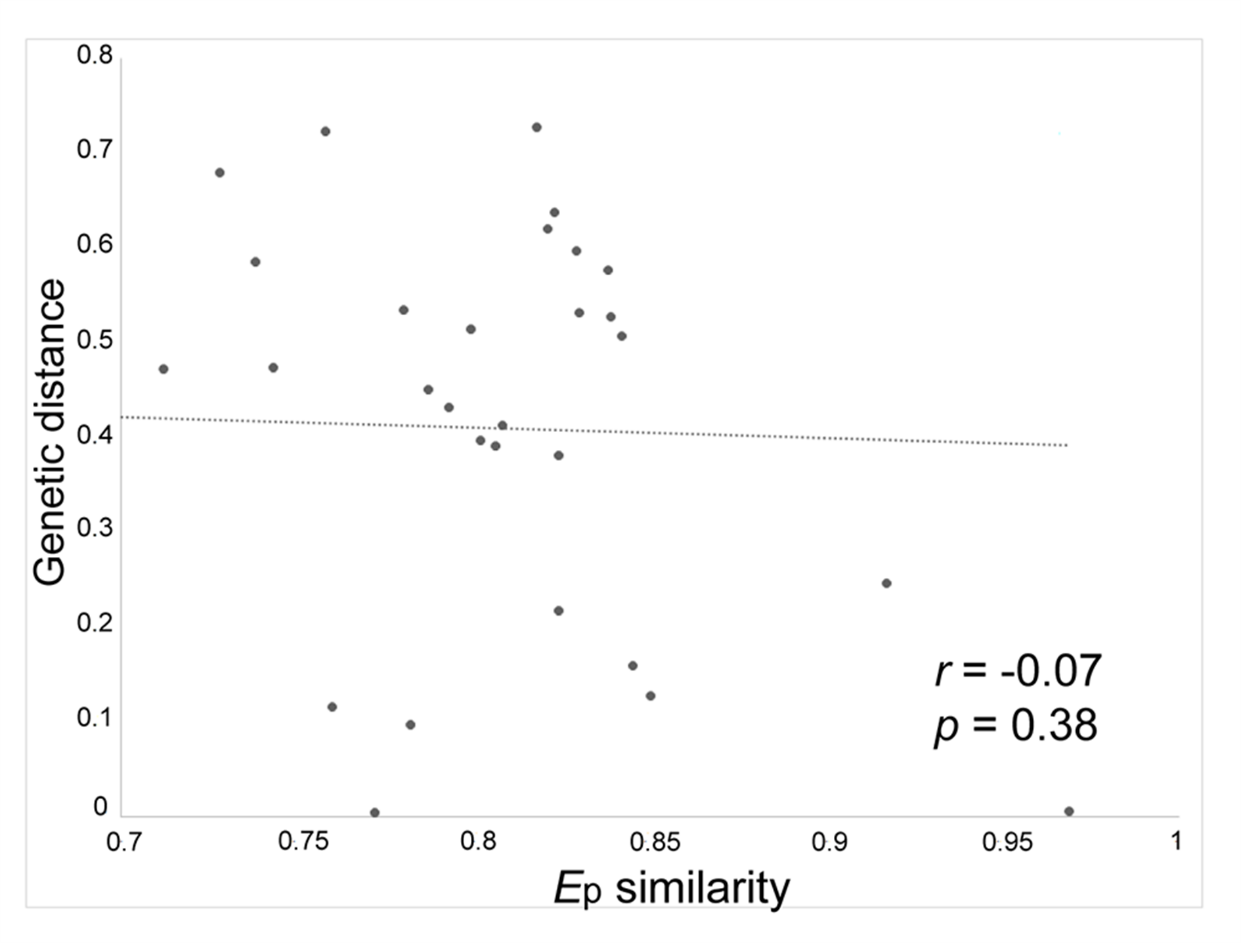

Supplement: Supplementary file 10 — Additional file 10 The relationship between population expression similarity (Ep similarity) and genetic distance. [file 12864_2021_7682_MOESM10_ESM.docx]
