# Supplementary material for: Population transcriptomic sequencing reveals allopatric divergence and local adaptation in Pseudotaxus chienii (Taxaceae)
Source: BMC Genomics. 2021 May 26;22:388. doi: 10.1186/s12864-021-07682-3 (PMC8157689; doi:10.1186/s12864-021-07682-3)
Supplement: Supplementary file 11 — Additional file 11. Independent contributions of five bioclimatic variables for four groups. [file 12864_2021_7682_MOESM11_ESM.docx]

**Additional file 11.** Independent contributions of five bioclimatic variables for four groups.

| **Group** | **Bio11** | **Bio13** | **Bio14** | **Bio15** | **Bio18** |
| --- | --- | --- | --- | --- | --- |
| GX | 64.87% | 0.50% | 21.49% | 0.29% | 12.85% |
| HN | 4.69% | 0.37% | 4.70% | 73.24% | 17.00% |
| JX | 0.56% | 17.38% | 46.56% | 35.00% | 0.51% |
| ZJ | 22.03% | 26.83% | 28.45% | 17.04% | 5.64% |

JX, Jiangxi group; ZJ, Zhejiang group; GX, Guangxi group; HN, Hunan group; Bio11, mean temperature of the coldest quarter; Bio13, precipitation of the wettest month; Bio14, precipitation of the driest month; Bio15, precipitation seasonality (CV); Bio18, precipitation of the warmest quarter.
