# Supplementary material for: Population transcriptomic sequencing reveals allopatric divergence and local adaptation in Pseudotaxus chienii (Taxaceae)
Source: BMC Genomics. 2021 May 26;22:388. doi: 10.1186/s12864-021-07682-3 (PMC8157689; doi:10.1186/s12864-021-07682-3)

**Additional file 12.** Predicted potential distributions for the four groups of *Pseudotaxus chienii* (ZJ, JX, GX, and HN). (a) Present day. (b) Last interglacial (LIG, c. 140–120 kya). (c) Last glacial maximum (LGM, c. 21 kya). (d) Future (2050, average for 2041–2060).


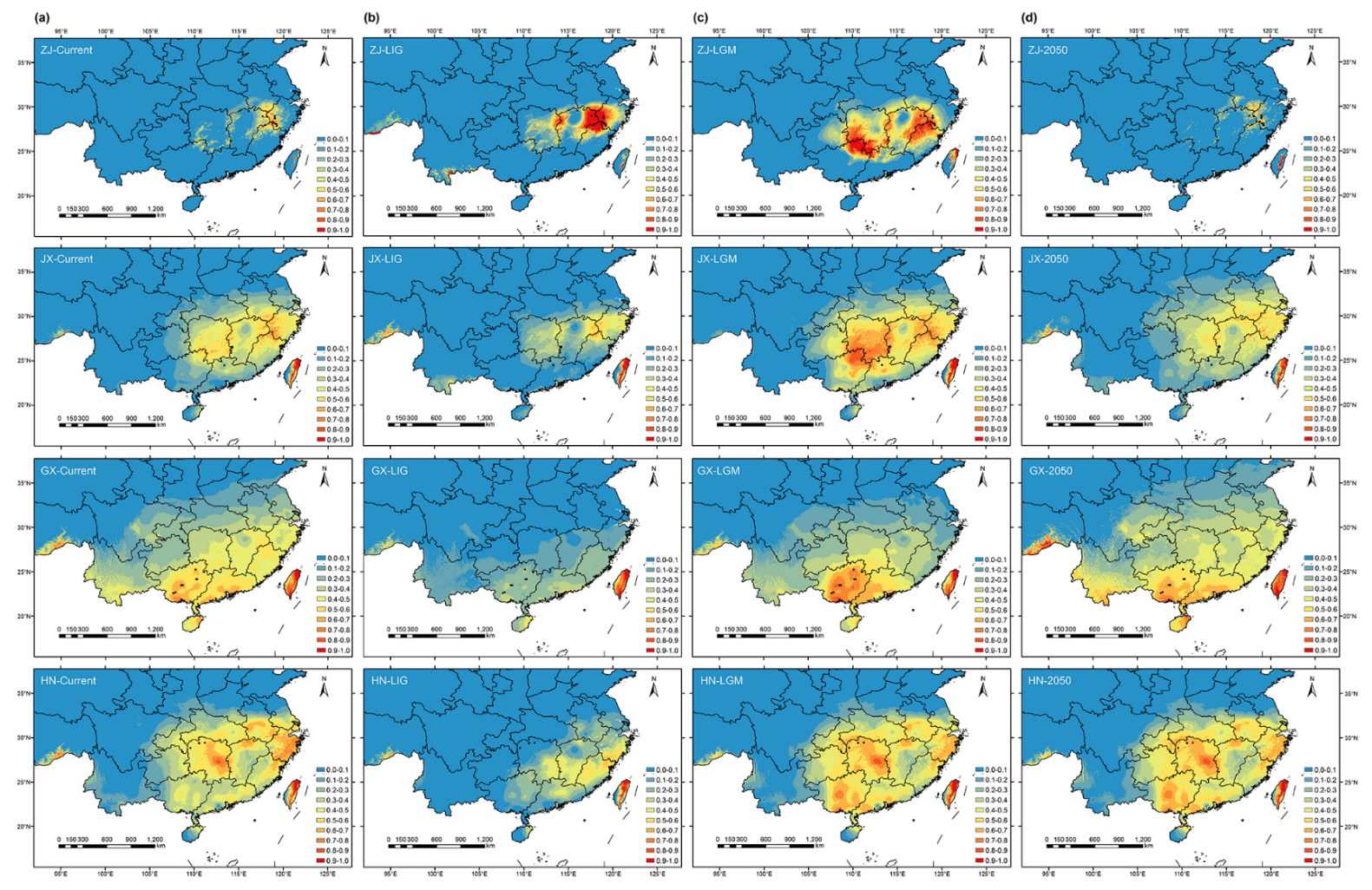

Supplement: Supplementary file 12 — Additional file 12 Predicted potential distributions for the four groups of Pseudotaxus chienii (ZJ, JX, GX, and HN). (a) Present day. (b) Last interglacial (LIG, c. 120–140 kya). (c) Last glacial maximum (LGM, c. 21 kya). (d) Future (2050, average for 2041–2060). [file 12864_2021_7682_MOESM12_ESM.docx]
