# Supplementary material for: Population transcriptomic sequencing reveals allopatric divergence and local adaptation in Pseudotaxus chienii (Taxaceae)
Source: BMC Genomics. 2021 May 26;22:388. doi: 10.1186/s12864-021-07682-3 (PMC8157689; doi:10.1186/s12864-021-07682-3)

**Additional file 14.** Gene ontology (GO) annotation of the candidate unigenes containing outlier SNPs identified in BayeScan.


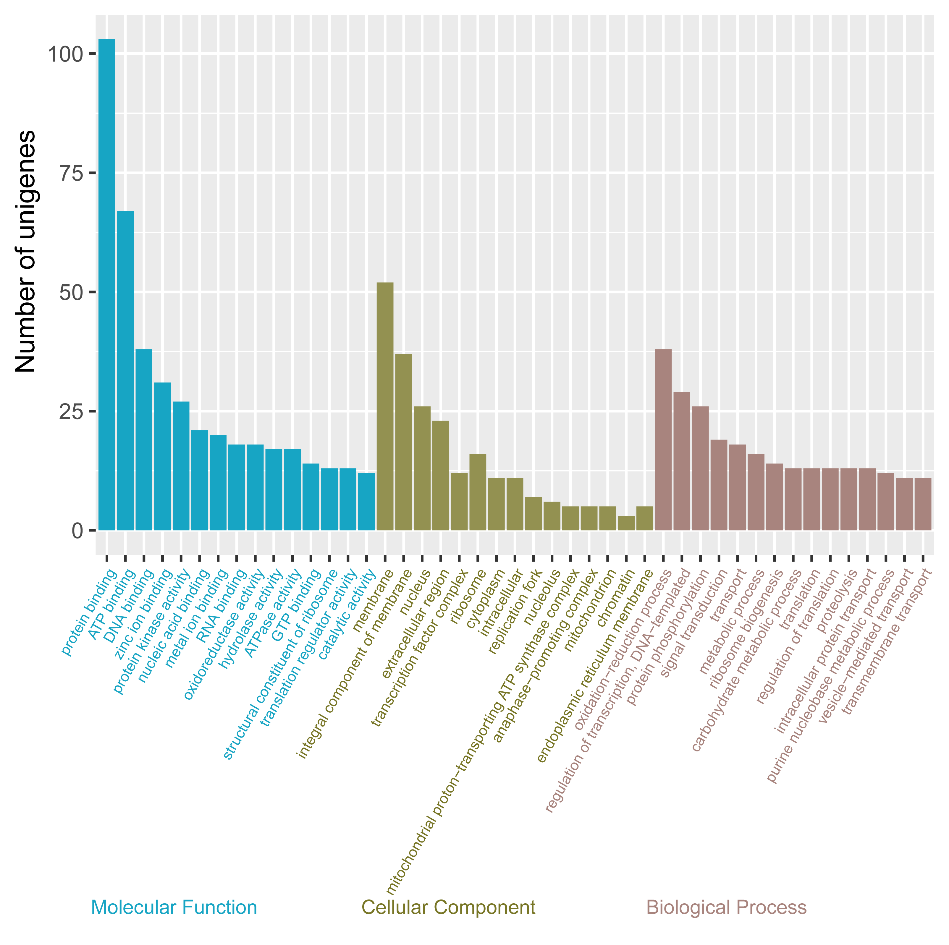

Supplement: Supplementary file 14 — Additional file 14. Gene ontology (GO) annotation of the candidate unigenes containing outlier SNPs identified in BayeScan. [file 12864_2021_7682_MOESM14_ESM.docx]
