# Supplementary material for: Population transcriptomic sequencing reveals allopatric divergence and local adaptation in Pseudotaxus chienii (Taxaceae)
Source: BMC Genomics. 2021 May 26;22:388. doi: 10.1186/s12864-021-07682-3 (PMC8157689; doi:10.1186/s12864-021-07682-3)
Supplement: Supplementary file 17 — Additional file 17. Kyoto Encyclopedia of Genes and Genomes (KEGG) pathway enrichment analysis of the 87 candidate unigenes for habitat adaptation in the Zhejiang (ZJ) group. [file 12864_2021_7682_MOESM17_ESM.docx]

**Additional file 17.** Kyoto Encyclopedia of Genes and Genomes (KEGG) pathway enrichment analysis of the 87 candidate unigenes for habitat adaptation in the Zhejiang (ZJ) group.

**
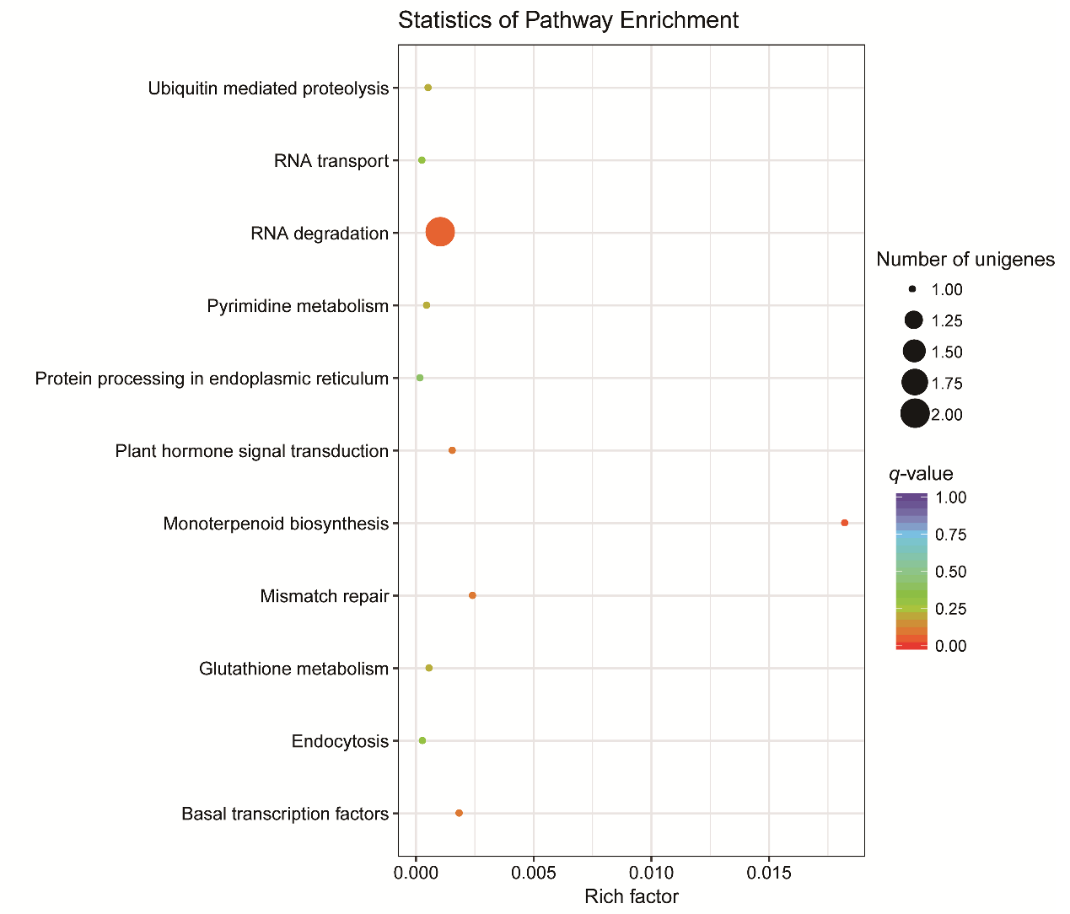
**
