# Supplementary material for: Population transcriptomic sequencing reveals allopatric divergence and local adaptation in Pseudotaxus chienii (Taxaceae)
Source: BMC Genomics. 2021 May 26;22:388. doi: 10.1186/s12864-021-07682-3 (PMC8157689; doi:10.1186/s12864-021-07682-3)
Supplement: Supplementary file 19 — Additional file 19. Kyoto Encyclopedia of Genes and Genomes (KEGG) pathway enrichment analysis of three candidate unigenes for habitat adaptation in the Hunan (HN) group. [file 12864_2021_7682_MOESM19_ESM.docx]

**Additional file 19.** Kyoto Encyclopedia of Genes and Genomes (KEGG) pathway enrichment analysis of three candidate unigenes for habitat adaptation in the Hunan (HN) group.

**
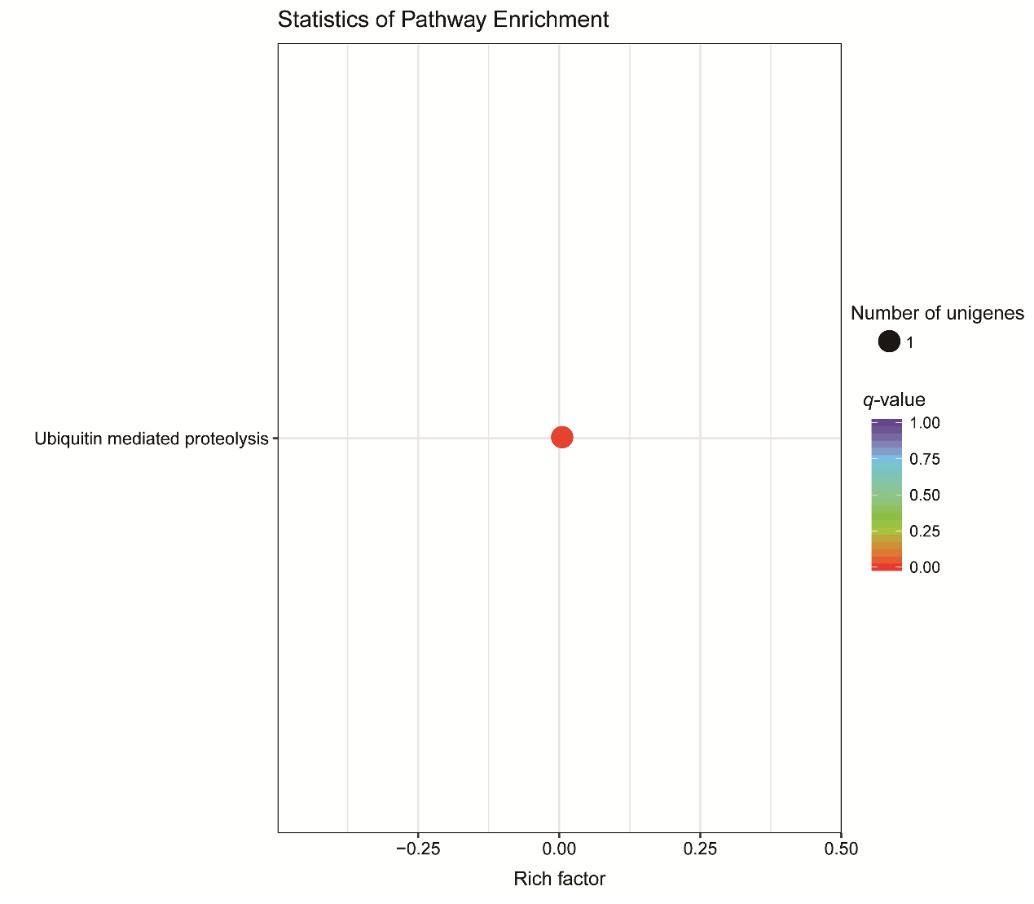
**
