# Supplementary material for: Population transcriptomic sequencing reveals allopatric divergence and local adaptation in Pseudotaxus chienii (Taxaceae)
Source: BMC Genomics. 2021 May 26;22:388. doi: 10.1186/s12864-021-07682-3 (PMC8157689; doi:10.1186/s12864-021-07682-3)
Supplement: Supplementary file 21 — Additional file 21. The significant environmental and geographic variables retained by the initial step-forward selection method for all loci and outlier loci. [file 12864_2021_7682_MOESM21_ESM.docx]

**Additional file 21.** The significant environmental and geographic variables retained by the initial step-forward selection method for all loci and outlier loci.

| **All loci** | | | | **Outlier loci** | | | |
| --- | --- | --- | --- | --- | --- | --- | --- |
| **Variable** | **Adjusted *R*^2^** | ***F*** | ***p*-value** | **Variable** | **Adjusted *R*^2^** | ***F*** | ***p*-value** |
| Environmental variables | | | | Environmental variables | | | |
| Bio11 | 0.31511 | 3.3628 | 0.001 | Bio11 | 0.64056 | 7.9914 | 0.001 |
| Aspect | 0.29893 | 4.2794 | 0.001 | Fe | 0.61543 | 9.1072 | 0.001 |
| Fe | 0.27617 | 4.5792 | 0.001 | Cu | 0.58456 | 10.285 | 0.001 |
| Bio14 | 0.25077 | 4.5487 | 0.001 | Bio14 | 0.54675 | 10.2402 | 0.001 |
| LAI | 0.22495 | 4.6732 | 0.001 | Altitude | 0.50609 | 10.3572 | 0.001 |
| Altitude | 0.19758 | 4.6537 | 0.001 | Pb | 0.46165 | 14.5568 | 0.001 |
| Mg | 0.16966 | 9.7827 | 0.001 | Bio15 | 0.39214 | 36.6232 | 0.001 |
| Bio15 | 0.10086 | 13.0024 | 0.001 | Mg | 0.18786 | 25.7502 | 0.001 |
| Geographic variables | | | | Geographic variables | | | |
| dbMEM4 | 0.23903 | 3.4890 | 0.001 | dbMEM4 | 0.53542 | 6.0776 | 0.001 |
| dbMEM3 | 0.22064 | 4.1494 | 0.001 | dbMEM6 | 0.51229 | 5.7942 | 0.001 |
| dbMEM5 | 0.19703 | 4.8864 | 0.001 | dbMEM3 | 0.48959 | 6.9224 | 0.001 |
| dbMEM2 | 0.16731 | 9.5294 | 0.001 | dbMEM5 | 0.46053 | 10.6181 | 0.001 |
| dbMEM1 | 0.10031 | 12.9301 | 0.001 | dbMEM2 | 0.41111 | 22.5486 | 0.001 |
|  |  |  |  | dbMEM1 | 0.29139 | 45.0007 | 0.004 |

Fe, soil Fe content; Mg, soil Mg content; Cu, soil Cu content; Pb, soil Pb content; Bio11, mean temperature of the coldest quarter; Bio14, precipitation of the driest month; Bio15, precipitation seasonality (CV); LAI, leaf area index.
