# Supplementary material for: Population transcriptomic sequencing reveals allopatric divergence and local adaptation in Pseudotaxus chienii (Taxaceae)
Source: BMC Genomics. 2021 May 26;22:388. doi: 10.1186/s12864-021-07682-3 (PMC8157689; doi:10.1186/s12864-021-07682-3)
Supplement: Supplementary file 22 — Additional file 22. Outlier SNPs associated with environmental variables identified using LFMM. [file 12864_2021_7682_MOESM22_ESM.docx]

**Additional file 22.** Outlier SNPs associated with environmental variables identified using LFMM.

| **Unigenes** | **Position** | **Environmental variables** | | | |
| --- | --- | --- | --- | --- | --- |
| Cluster-24249 6.94002 | 2050 | FAPAR | PTC |  |  |
| Cluster-242496.100182 | 2172 | Bio13 | FAPAR | PTC |  |
| Cluster-242496.100509 | 570 | Bio11 | FAPAR |  |  |
| Cluster-242496.101765 | 2634 | Bio14 |  |  |  |
| Cluster-242496.102039 | 1451 | Pb |  |  |  |
| Cluster-242496.102159 | 5288 | Bio14 |  |  |  |
| Cluster-242496.102614 | 1140 | Zn |  |  |  |
| Cluster-242496.103204 | 362 | Aspect |  |  |  |
| Cluster-242496.103547 | 2277 | FAPAR |  |  |  |
| Cluster-242496.104498 | 185 | Bio13 |  |  |  |
| Cluster-242496.104565 | 709 | Zn |  |  |  |
| Cluster-242496.10469 | 142 | FAPAR | PTC | Pb | Slope |
| Cluster-242496.10469 | 123 | FAPAR | PTC | Pb | Slope |
| Cluster-242496.104746 | 2278 | Zn |  |  |  |
| Cluster-242496.105072 | 569 | Mn |  |  |  |
| Cluster-242496.105221 | 1388 | FAPAR |  |  |  |
| Cluster-242496.105412 | 727 | Cu |  |  |  |
| Cluster-242496.105990 | 319 | Bio11 | Cu |  |  |
| Cluster-242496.106365 | 3220 | Altitude |  |  |  |
| Cluster-242496.106600 | 2679 | Slope |  |  |  |
| Cluster-242496.106820 | 5670 | EVI |  |  |  |
| Cluster-242496.107089 | 3183 | FAPAR | Mg | Zn | Cu |
| Cluster-242496.107652 | 3449 | Bio14 | FAPAR | Mg | Slope |
| Cluster-242496.107652 | 723 | Bio14 | Mg | Cu | Slope |
| Cluster-242496.107652 | 3652 | Cu |  |  |  |
| Cluster-242496.107721 | 1951 | Aspect |  |  |  |
| Cluster-242496.107721 | 1921 | Aspect |  |  |  |
| Cluster-242496.108373 | 653 | Bio13 |  |  |  |
| Cluster-242496.108429 | 2003 | Slope |  |  |  |
| Cluster-242496.108939 | 1608 | FAPAR | Aspect |  |  |
| Cluster-242496.109050 | 583 | Slope |  |  |  |
| Cluster-242496.109050 | 584 | Slope |  |  |  |
| Cluster-242496.109238 | 2722 | PTC |  |  |  |
| Cluster-242496.109416 | 5151 | FAPAR |  |  |  |
| Cluster-242496.109416 | 5114 | FAPAR |  |  |  |
| Cluster-242496.109416 | 1248 | FAPAR |  |  |  |
| Cluster-242496.109439 | 2761 | Bio13 |  |  |  |
| Cluster-242496.109439 | 2663 | Bio13 |  |  |  |
| Cluster-242496.109439 | 2665 | Bio13 |  |  |  |
| Cluster-242496.109439 | 2776 | Bio13 |  |  |  |
| Cluster-242496.110091 | 179 | FAPAR |  |  |  |
| Cluster-242496.110101 | 272 | Cu |  |  |  |
| Cluster-242496.110902 | 1089 | Zn | Cu |  |  |
| Cluster-242496.110902 | 1020 | Zn | Cu |  |  |
| Cluster-242496.110902 | 1082 | Zn | Cu |  |  |
| Cluster-242496.110902 | 1046 | Zn | Cu |  |  |
| Cluster-242496.110902 | 958 | Zn |  |  |  |
| Cluster-242496.110902 | 959 | Zn |  |  |  |
| Cluster-242496.110902 | 1251 | Zn | Cu |  |  |
| Cluster-242496.110902 | 1373 | Zn |  |  |  |
| Cluster-242496.111070 | 1903 | Mg | Cu |  |  |
| Cluster-242496.111222 | 2018 | FAPAR | Aspect |  |  |
| Cluster-242496.111222 | 2016 | FAPAR | Aspect |  |  |
| Cluster-242496.111721 | 320 | Aspect |  |  |  |
| Cluster-242496.112085 | 1140 | Bio14 |  |  |  |
| Cluster-242496.112134 | 599 | FAPAR |  |  |  |
| Cluster-242496.112665 | 722 | FAPAR | PTC |  |  |
| Cluster-242496.112665 | 757 | FAPAR | PTC |  |  |
| Cluster-242496.113214 | 1086 | FAPAR |  |  |  |
| Cluster-242496.113581 | 2636 | LAI | PTC |  |  |
| Cluster-242496.113906 | 4059 | Pb |  |  |  |
| Cluster-242496.114622 | 515 | Aspect |  |  |  |
| Cluster-242496.114721 | 249 | LAI |  |  |  |
| Cluster-242496.114750 | 806 | Bio18 | Altitude |  |  |
| Cluster-242496.114750 | 1159 | Mn |  |  |  |
| Cluster-242496.114750 | 247 | Slope |  |  |  |
| Cluster-242496.115332 | 2091 | Bio11 |  |  |  |
| Cluster-242496.115607 | 1243 | Bio18 |  |  |  |
| Cluster-242496.116884 | 692 | Bio11 | Cu |  |  |
| Cluster-242496.117078 | 860 | Mg | Cu |  |  |
| Cluster-242496.117078 | 902 | Mg | Cu |  |  |
| Cluster-242496.117393 | 1112 | Aspect |  |  |  |
| Cluster-242496.117460 | 2112 | Bio14 |  |  |  |
| Cluster-242496.117805 | 724 | Altitude |  |  |  |
| Cluster-242496.118071 | 2336 | FAPAR | PTC | Pb | Slope |
| Cluster-242496.118388 | 2776 | Cu |  |  |  |
| Cluster-242496.118691 | 1871 | Bio13 |  |  |  |
| Cluster-242496.118841 | 1701 | LAI | Cu |  |  |
| Cluster-242496.119209 | 1713 | Bio18 |  |  |  |
| Cluster-242496.119585 | 962 | Pb |  |  |  |
| Cluster-242496.119843 | 60 | Bio18 | Altitude |  |  |
| Cluster-242496.119843 | 484 | Bio18 | Altitude |  |  |
| Cluster-242496.120336 | 832 | Bio14 |  |  |  |
| Cluster-242496.120336 | 573 | Bio14 |  |  |  |
| Cluster-242496.120362 | 296 | Bio13 |  |  |  |
| Cluster-242496.120646 | 5041 | PTC | Fe |  |  |
| Cluster-242496.121328 | 289 | FAPAR | PTC | Mg |  |
| Cluster-242496.122480 | 699 | PTC | Pb |  |  |
| Cluster-242496.122718 | 48 | Aspect |  |  |  |
| Cluster-242496.124284 | 365 | Bio14 |  |  |  |
| Cluster-242496.126657 | 1399 | Pb |  |  |  |
| Cluster-242496.126932 | 2958 | Bio13 | Bio18 |  |  |
| Cluster-242496.126932 | 2800 | Bio18 | Altitude |  |  |
| Cluster-242496.126932 | 3033 | Bio18 | Altitude |  |  |
| Cluster-242496.138898 | 1523 | Bio13 |  |  |  |
| Cluster-242496.55130 | 537 | Mg |  |  |  |
| Cluster-242496.55130 | 1057 | Zn |  |  |  |
| Cluster-242496.59179 | 1010 | Mg |  |  |  |
| Cluster-242496.63217 | 1395 | Bio13 |  |  |  |
| Cluster-242496.66913 | 2710 | Cu |  |  |  |
| Cluster-242496.69003 | 614 | Mn |  |  |  |
| Cluster-242496.71599 | 1237 | Altitude |  |  |  |
| Cluster-242496.72284 | 544 | Cu |  |  |  |
| Cluster-242496.73323 | 2133 | FAPAR |  |  |  |
| Cluster-242496.74400 | 1412 | Altitude |  |  |  |
| Cluster-242496.74947 | 356 | Mg | Zn | Cu |  |
| Cluster-242496.74947 | 294 | Mg | Zn | Cu |  |
| Cluster-242496.75238 | 2785 | Bio18 |  |  |  |
| Cluster-242496.75754 | 1866 | FAPAR | Aspect |  |  |
| Cluster-242496.76566 | 2119 | LAI |  |  |  |
| Cluster-242496.77376 | 3183 | FAPAR | PTC |  |  |
| Cluster-242496.78341 | 915 | Bio18 |  |  |  |
| Cluster-242496.78746 | 1227 | Mg | Zn | Cu |  |
| Cluster-242496.79401 | 1295 | Zn |  |  |  |
| Cluster-242496.79401 | 1371 | Zn |  |  |  |
| Cluster-242496.79670 | 577 | Mn |  |  |  |
| Cluster-242496.79743 | 1511 | FAPAR | Slope |  |  |
| Cluster-242496.79743 | 1088 | FAPAR |  |  |  |
| Cluster-242496.80576 | 2252 | Bio18 |  |  |  |
| Cluster-242496.80895 | 1822 | Slope |  |  |  |
| Cluster-242496.82443 | 1053 | Zn |  |  |  |
| Cluster-242496.82554 | 2823 | Bio11 |  |  |  |
| Cluster-242496.83212 | 269 | FAPAR | PTC | Slope |  |
| Cluster-242496.84223 | 97 | Cu | Aspect |  |  |
| Cluster-242496.84378 | 1469 | Cu |  |  |  |
| Cluster-242496.84580 | 937 | Bio18 |  |  |  |
| Cluster-242496.84971 | 540 | Zn |  |  |  |
| Cluster-242496.85250 | 803 | FAPAR | LAI | Mg | Cu |
| Cluster-242496.85592 | 1216 | Cu |  |  |  |
| Cluster-242496.85724 | 3158 | Aspect |  |  |  |
| Cluster-242496.85734 | 351 | Pb |  |  |  |
| Cluster-242496.85941 | 1157 | LAI | PTC |  |  |
| Cluster-242496.86298 | 1279 | Bio14 |  |  |  |
| Cluster-242496.87122 | 968 | Mn |  |  |  |
| Cluster-242496.87131 | 2144 | FAPAR |  |  |  |
| Cluster-242496.87131 | 317 | Mn |  |  |  |
| Cluster-242496.88268 | 1534 | Altitude |  |  |  |
| Cluster-242496.88306 | 990 | Aspect |  |  |  |
| Cluster-242496.88619 | 1740 | Altitude |  |  |  |
| Cluster-242496.89067 | 160 | Bio14 |  |  |  |
| Cluster-242496.89391 | 287 | Pb |  |  |  |
| Cluster-242496.89475 | 1921 | Bio14 |  |  |  |
| Cluster-242496.90372 | 1975 | Aspect |  |  |  |
| Cluster-242496.90593 | 1757 | FAPAR | PTC | Aspect |  |
| Cluster-242496.90593 | 1742 | FAPAR | PTC | Aspect |  |
| Cluster-242496.91009 | 1036 | Aspect |  |  |  |
| Cluster-242496.91061 | 1031 | FAPAR | Slope |  |  |
| Cluster-242496.91061 | 1134 | FAPAR | PTC |  |  |
| Cluster-242496.91061 | 1007 | FAPAR | PTC |  |  |
| Cluster-242496.91379 | 1044 | Bio14 |  |  |  |
| Cluster-242496.91392 | 476 | Pb |  |  |  |
| Cluster-242496.91513 | 2481 | Mg | Zn | Cu |  |
| Cluster-242496.92123 | 1028 | Zn | Cu |  |  |
| Cluster-242496.92123 | 1001 | Zn |  |  |  |
| Cluster-242496.93052 | 116 | Bio18 | Mn |  |  |
| Cluster-242496.93274 | 1711 | PTC |  |  |  |
| Cluster-242496.94047 | 514 | FAPAR | LAI | PTC |  |
| Cluster-242496.95401 | 727 | Zn |  |  |  |
| Cluster-242496.95401 | 700 | Zn |  |  |  |
| Cluster-242496.95685 | 1928 | Bio18 |  |  |  |
| Cluster-242496.95885 | 302 | Bio14 | Cu |  |  |
| Cluster-242496.96982 | 760 | Bio14 | Mg |  |  |
| Cluster-242496.96982 | 4378 | Bio14 |  |  |  |
| Cluster-242496.99779 | 317 | Bio13 |  |  |  |

Bio11, mean temperature of the coldest quarter; Bio13, precipitation of the wettest month; Bio14, precipitation of the driest month; Bio18, precipitation of the warmest quarter; NDVI, normalized difference vegetation index; PTC, percent tree cover; LAI, leaf area index; EVI, enhanced vegetation index; FAPAR, fraction of absorbed photosynthetically active radiation.
