# Supplementary material for: Population transcriptomic sequencing reveals allopatric divergence and local adaptation in Pseudotaxus chienii (Taxaceae)
Source: BMC Genomics. 2021 May 26;22:388. doi: 10.1186/s12864-021-07682-3 (PMC8157689; doi:10.1186/s12864-021-07682-3)
Supplement: Supplementary file 24 — Additional file 24 The environmental variables with variance inflation factors (VIFs) < 10 for 10 Pseudotaxus chienii populations. [file 12864_2021_7682_MOESM24_ESM.docx]

**Additional file 24.** The environmental variables with variance inflation factors (VIFs) < 10 for 10 *Pseudotaxus chienii* populations.

| **Population** | **Bio11** | **Bio13** | **Bio14** | **Bio15** | **Bio18** | **EVI** | **FAPAR** | **LAI** | **PTC** | **Altitude** | **Aspect** | **Slope** | **Mg** | **Fe** | **Mn** | **Zn** | **Cu** | **Pb** |
| --- | --- | --- | --- | --- | --- | --- | --- | --- | --- | --- | --- | --- | --- | --- | --- | --- | --- | --- |
| BJS | 40 | 271 | 51 | 48 | 578 | 0.56 | 0.99 | 6.73 | 68.61 | 1293 | 75.29 | 33.54 | 4.90 | 11.29 | 0.23 | 0.74 | 0.02 | 0.04 |
| ZZB | 22 | 303 | 54 | 50 | 642 | 0.61 | 0.95 | 6.06 | 64.33 | 1297 | 65.83 | 22.31 | 9.05 | 13.38 | 0.37 | 0.89 | 0.02 | 0.09 |
| SQS | 32 | 345 | 53 | 52 | 732 | 0.49 | 0.96 | 6.00 | 65.17 | 1343 | 179.07 | 22.17 | 3.93 | 16.57 | 0.34 | 0.53 | 0.03 | 0.07 |
| DXG | 29 | 337 | 47 | 49 | 792 | 0.54 | 0.97 | 6.42 | 69.94 | 1487 | 351.04 | 12.17 | 1.69 | 23.47 | 0.14 | 0.14 | 0.01 | 0.06 |
| LMD | 39 | 319 | 47 | 50 | 664 | 0.55 | 0.99 | 6.78 | 72.67 | 1049 | 255.36 | 17.21 | 1.88 | 12.49 | 0.84 | 0.28 | 0.01 | 0.07 |
| MS | 59 | 331 | 45 | 52 | 678 | 0.55 | 0.95 | 6.01 | 58.72 | 1158 | 147.54 | 26.66 | 1.14 | 44.54 | 0.17 | 0.24 | 0.01 | 0.07 |
| SMJ | 51 | 308 | 47 | 52 | 603 | 0.61 | 0.94 | 6.66 | 63.72 | 914 | 272.45 | 29.59 | 1.61 | 20.72 | 0.31 | 0.36 | 0.01 | 0.08 |
| LHS | 85 | 279 | 40 | 64 | 683 | 0.50 | 0.94 | 6.42 | 60.67 | 1080 | 296.43 | 21.32 | 2.88 | 10.55 | 0.22 | 0.51 | 0.01 | 0.05 |
| YSGY | 65 | 286 | 40 | 64 | 717 | 0.56 | 0.99 | 6.86 | 74.56 | 1182 | 1.40 | 4.22 | 1.57 | 3.41 | 0.09 | 0.27 | 0.01 | 0.04 |
| ZJJ | 36 | 230 | 35 | 54 | 601 | 0.58 | 0.96 | 6.07 | 54.11 | 1002 | 35.58 | 11.29 | 4.24 | 24.66 | 0.39 | 1.14 | 0.02 | 0.09 |

Bio11, mean temperature of the coldest quarter; Bio13, precipitation of the wettest month; Bio14, precipitation of the driest month; Bio15, precipitation seasonality (CV); Bio18, precipitation of the warmest quarter; NDVI, normalized difference vegetation index; PTC, percent tree cover; LAI, leaf area index; EVI, enhanced vegetation index; FAPAR, fraction of absorbed photosynthetically active radiation.
